# Probing decoherence in molecular 4f qubits†

**DOI:** 10.1039/d4sc05304d

**Published:** 2024-10-30

**Authors:** Steen H. Hansen, Christian D. Buch, Jonatan B. Petersen, Michelle Rix, Marc Ubach I Cervera, Asger Strandfelt, Richard E. P. Winpenny, Eric J. L. McInnes, Stergios Piligkos

**Affiliations:** a Department of Chemistry, University of Copenhagen DK-2100 Copenhagen Denmark piligkos@chem.ku.dk; b Department of Chemistry, School of Natural Science, The University of Manchester Oxford Road Manchester M13 9PL UK

## Abstract

We probe herein the fundamental factors that induce decoherence in ensembles of molecular magnetic materials. This is done by pulse Electron Paramagnetic Resonance measurements at X-band (∼9.6 GHz) on single crystals of Gd@Y(trensal) at 0.5, 10^−1^, 10^−2^ and 10^−3^% doping levels, using Hahn echo, partial refocusing and CPMG sequences. The phase memory time, *T*_m_, obtained by the Hahn echo sequence at X-band is compared to the one previously determined at higher frequency/magnetic field (∼240 GHz). The combined information from these experiments allows to gain insight into the contributions to decoherence originating from various relaxation mechanisms such as spin–lattice relaxation, electron and nuclear spin diffusion and instantaneous diffusion. We show that while at high magnetic fields *T*_m_ is limited by spin–lattice relaxation seemingly attributed to a direct process, at lower fields the limiting factor is spectral diffusion. At X-band, for Gd@Y(trensal) we determine a *T*_m_ in the range 1–12 μs, at 5 K, depending on the magnetic field and concentration of Gd(trensal) in the isostructural diamagnetic host Y(trensal). Importantly, Gd@Y(trensal) displays measurable coherence at temperatures above liquid nitrogen ones, with 125 K being the upper limit. At the lowest dilution level of **10^−3^%** and under dynamic decoupling conditions, the ratio of *T*_m_*versus* the time it takes to implement a quantum gate, *T*_G_, reaches the order of 10^4^, in the example of a single qubit π-rotation, which corresponds to an upper limit of gate fidelity of the order of 99.99%, reaching thus the lower limit of qubit figure of merit required for implementations in quantum information technologies.

## Abstract 2

We probe herein the fundamental factors that induce decoherence in ensembles of molecular magnetic materials.

## Graphical abstract

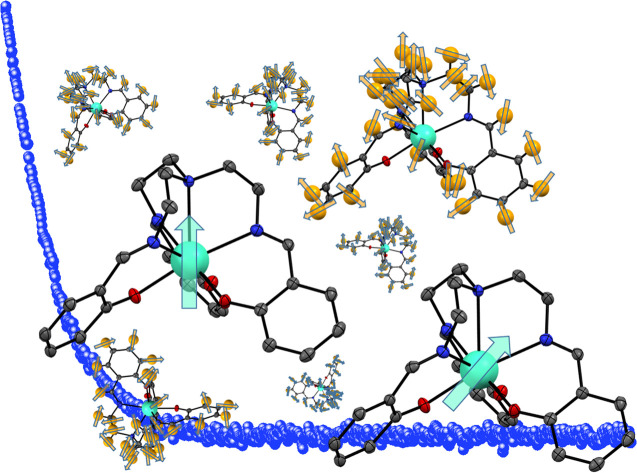

## Introduction

Coherent superposition and entanglement of quantum mechanical states are fundamental properties of matter the exploitation of which forms the basis for a variety of emerging new technologies, collectively referred to as quantum technologies,^1–3^ such as quantum computing,^4–8^ simulators,^9^ communications,^10^ sensing,^11–16^ metrology,^10^ cryptography^17^ and imaging.^18^ Two-dimensional or higher dimensionality quantum systems (qubits or qudits, respectively) such as superconducting nanostructures,^19,20^ photons,^21,22^ nitrogen vacancies in diamonds,^23–25^ single atoms in silicon,^26–28^ trapped ions,^29,30^ photo excited states,^31–33^ atoms on surfaces^34^ and metal complexes^35–57^ can be used as quantum hardware. Unlike many other candidates, molecular magnetic materials routinely display many low energy states compatible with the encoding of multiple qubits and qudits.^57–62^ Importantly, the multilevel structure of their eigenspectrum provides an expanded computational space dimension, as compared to individual qubits, and offers the capability to implement quantum algorithms at the single molecule level,^47,48,63^ efficiently encode quantum error correction algorithms^54,57,64^ or the first example of a quantum simulation using a molecular system.^53^ Implementation of single and entangled quantum gates on molecular magnetic materials is performed by coherent manipulation of individual or entangled electronic or electronuclear angular momenta.^65,66^ Such coherent manipulations are achieved by electromagnetic pulse excitations, usually within an applied external magnetic field. The phase memory time, *T*_m_, reflects the time for which the information encoding state retains its phase coherence.^67^ According to the DiVincenzo criteria,^68^ the fidelity with which such states are created depends on the relative magnitude of *T*_m_ and the time it takes to implement a quantum gate, *T*_G_, with the ratio *T*_m_/*T*_G_ required to be of the order of at least 10^4^ to achieve a satisfactory fidelity. Thus, *T*_m_ and *T*_G_ are critical parameters determining the suitability of a given material as quantum hardware. Decoherence,^69^ the loss of coherence, is due to the interaction of the quantum system with its environment and results in loss of superposition collapsing the dynamic state of the quantum system to its thermal equilibrium eigenstates. Decoherence is due to fluctuations of the resonance frequency of the quantum system, partly induced by local magnetic field fluctuations generated by the dynamics of environmental angular momenta, such as electronic or nuclear spins. Such fluctuations can be induced by the electromagnetic pulse excitation, resulting in instantaneous diffusion or by the intrinsic dynamics of the environment resulting in spectral diffusion. The main factors to which spectral diffusion is usually attributed to are electron and nuclear spin diffusion. However, the relative contributions of such local magnetic field fluctuations diminish with increasing resonance frequency. Thus, the phase memory time should increase at higher resonance frequencies, which in the case of real or effective spin-half systems translates to higher applied magnetic fields. However, spin–lattice relaxation, reflecting the longitudinal relaxation induced to the quantum system by exchange of angular momentum with the lattice phonons, parametrised by the inverse of the longitudinal relaxation time, *T*_1_, which is the upper limit for *T*_m_*via* the dependence *T*_m_ ≤ 2*T*_1_, increases with increasing external magnetic field, especially when determined by a direct process.^70,71^

We probe herein the relative importance of these different decoherence mechanisms to *T*_m_. We have previously studied Gd@Y(trensal) at the **0.5%** doping level in the isostructural diamagnetic host Y(trensal) (**0.5%**, Gd_0.005_Y_0.995_(trensal)), with H_3_trensal = 2,2′,2′′-tris(salicylideneimino)triethylamine)), by pulse Electron Paramagnetic Resonance (EPR) at high frequency (240 GHz, 9 T). These studies revealed one of the highest *T*_m_ (12 μs at 3 K) amongst the to date proposed 4f molecular qubits.^56^ However, under these conditions *T*_m_ is limited by a *T*_1_ of the order of 30 μs at 3 K, by what appears to be a direct process.^56^ SQUID magnetometry of **0.5%** at 0.3 T revealed a *T*_1_ of the order of 20 ms at 1.9 K (Fig. S1, S2 and Tables S1, S2†). Other derivatives of Gd(trensal) have been found to have a *T*_1_ of hundreds of ms in the magnetic field regime of 1000–5000 G.^72,73^ Thus, at magnetic fields of the order of the ones involved at X-band EPR (∼9.6 GHz), *T*_1_ increases by about 3 orders of magnitude with respect to the one observed at 9 T and so does the upper limit imposed to *T*_m_ by *T*_1_. We study herein, *T*_m_ and *T*_1_ of single crystals of Gd@Y(trensal) at **0.5%**, **10^−1^%**, **10^−2^%** and **10^−3^%** doping levels by X-band pulse EPR at magnetic fields in the range 0 to 0.7 T, to probe *T*_m_, and the factors that determine it, under these conditions.

## Experimental

### Sample preparation

Single crystals of Gd_0.005_Y_0.995_(trensal) (**0.5%**), Gd_0.001_Y_0.999_(trensal) (**10**^**−1**^**%**), Gd_0.0001_Y_0.9999_(trensal) (**10**^**−2**^**%**) and Gd_0.00001_Y_0.99999_(trensal) (**10**^**−3**^**%**) were grown according to the literature.^74^ All chemicals and solvents for the synthesis were obtained from commercial sources except for Gd(OTf)_3_·9H_2_O and Y(OTf)_3_·9H_2_O which were prepared from the corresponding oxide and triflic acid as described in the literature.^75^

### Electron paramagnetic resonance (EPR)

All pulse EPR measurements were performed on single crystals of Gd(trensal) doped into the isostructural diamagnetic host Y(trensal).

#### Measurements on 0.5%

Pulse EPR spectra were recorded at X-band on a Bruker Elexsys 580 spectrometer fitted with a Bruker ER 4118X-MD5 dielectric resonator. The setup was cooled with a Bruker Flexline Cryogen-free system and the temperature controlled with an Oxford Instruments Mercury ITC. A single crystal of **0.5%** was placed with the unique axis parallel to the magnetic field (*B*_0_ ∥ *C*_3_). The crystal alignment was checked by comparing echo-detected field-sweep (EDFS) measurements to a simulated spectrum (Fig. 2, top) based on the crystal field (CF) parameters determined in our previous study (Table S3†).^56^

#### Measurements on 10^−1^%, 10^−2^% and 10^−3^%

Pulse EPR spectra were recorded at X-band on a Bruker Elexsys 580 spectrometer fitted with a Bruker ER 4118X-MD5W dielectric resonator. The setup was cooled by an oxford instruments flow cryostat and the temperature controlled with an Oxford Instruments Mercury ITC. Because of their larger than 3 mm length, single crystals of **10**^**−3**^**%** were placed with the unique axis vertical, thus perpendicular to the magnetic field (*B*_0_ ⊥ *C*_3_), which is applied in the horizontal plane. EDFS spectra (Fig. 2, bottom for **10**^**−3**^**%**) were measured using a standard Hahn echo sequence π/2–*τ*–π–*τ*–echo with π-pulses of 32 ns and *τ* of 300 ns.

#### *T*_1_ and *T*_m_ measurements

*T*_1_ and *T*_m_ were measured using inversion recovery and Hahn-echo decay pulse sequences π–*t*–π/2–*τ*–π–*τ*–echo and π/2–*τ*–π–*τ*–echo, respectively. In the case of *T*_m_ measurements, a large degree of ESEEM was observed with a frequency of *ω* ≈ 1 MHz. At low temperatures, the ESEEM effect was suppressed by use of long pulses. Due to the decrease in *T*_m_ with increasing temperature, this was not possible at temperatures above 20 K. *T*_m_ decays were modelled as mono-exponential ones. *T*_1_ decays of **10**^**−3**^**%** were also modelled as mono-exponential ones, while those of **0.5%** required use of bi-exponential functions.

#### Partial refocusing pulses

Instantaneous diffusion was probed by use of a π/2–*τ*–*Θ*–*τ*–echo sequence,^67,76,77^ with 80 ns π/2 pulses and *Θ* pulses of 40, 80, 120 and 160 ns.

#### Carr-Purcell-Meiboom-Gill (CPMG) sequence

Relaxation traces using CPMG dynamic refocusing,^67,78,79^ consisting of the sequence: *π*_*x*_/2–[*τ*–π_*y*_–*τ*]_*n*_–echo, with *n* up to 120, were measured using π/2 pulse lengths of 16 ns.

## Results and discussion

**Fig. 1:**
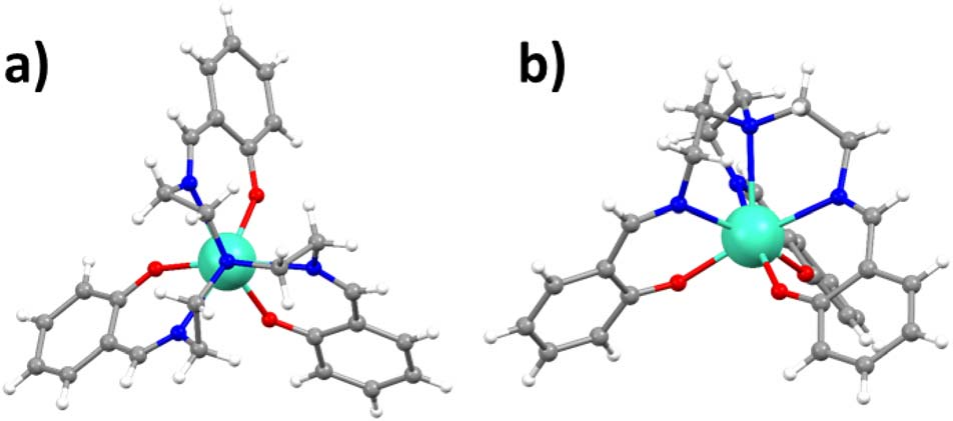
Molecular structure of Gd(trensal) (a) seen along the threefold symmetry axis and (b) seen from the side. Colour code: C, gray; H, white; N, nitrogen; O, oxygen; Gd, cyan.

**Fig. 2:**
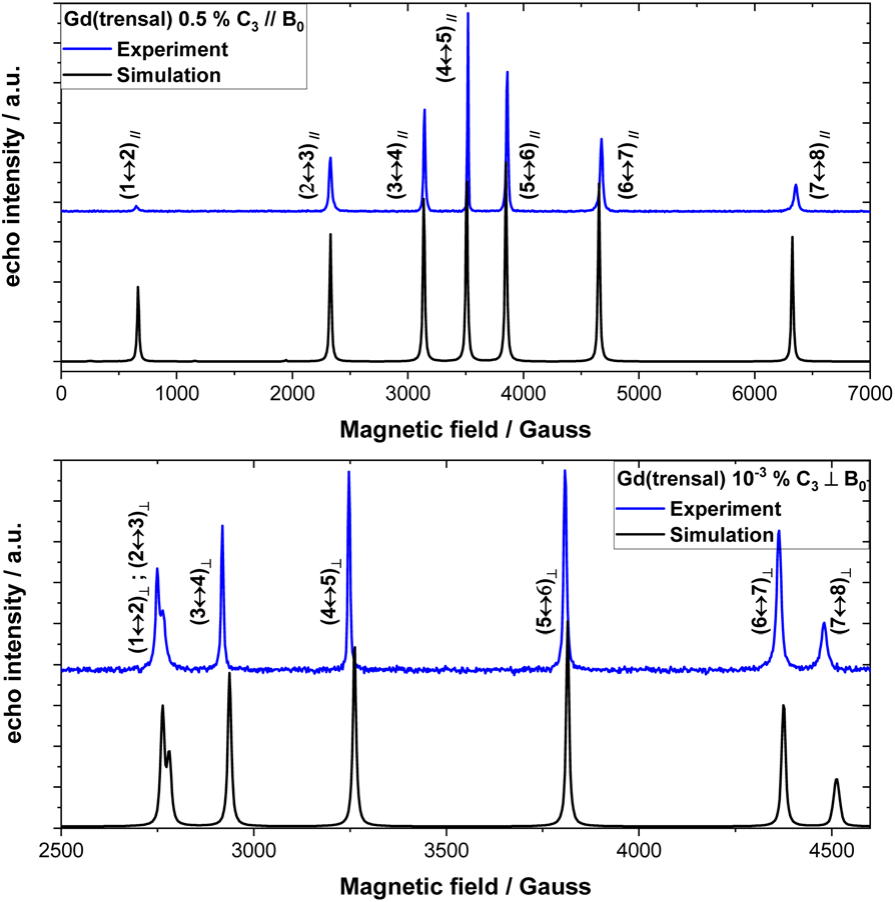
EDFS (blue) of **0.5%** with *B*_0_ ∥ *C*_3_ (top) and of **10**^**−3**^**%** with *B*_0_ ⊥ *C*_3_ (bottom) at 5.5 K, compared to simulations (black) using previously determined CF parameters (Table S3†).

Gd(trensal), as other members of the Ln(trensal) family,^80–82^ crystallises in the trigonal space group *P*3̄*c*1. The Gd(iii) centre is hepta-coordinated by the trensal ligand, with the ligand forming a three-armed chiral screw around the metal centre (Fig. 1). In the crystal structure, the crystallographic and molecular *C*_3_ axes coincide. They also coincide with the hexagonal axis of the macroscopic crystal. A single crystal of Gd(trensal) was oriented parallel or perpendicular to the magnetic field, *B*_0_. The EDFS spectrum of **0.5%** with *B*_0_ ∥ *C*_3_ shows the expected seven allowed EPR transitions of the ^8^S_7/2_ term at the same magnetic fields as predicted by the simulation (Fig. 2, top, S3 and S4†). For **10**^**−3**^**%** and *B*_0_ ⊥ *C*_3_, only 6 transitions were observed as the transitions between levels 1–2, (1 ↔ 2)_⊥_, and 2–3, (2 ↔ 3)_⊥_, overlap (Fig. 2, bottom and S5).

### Relaxation

**Fig. 3:**
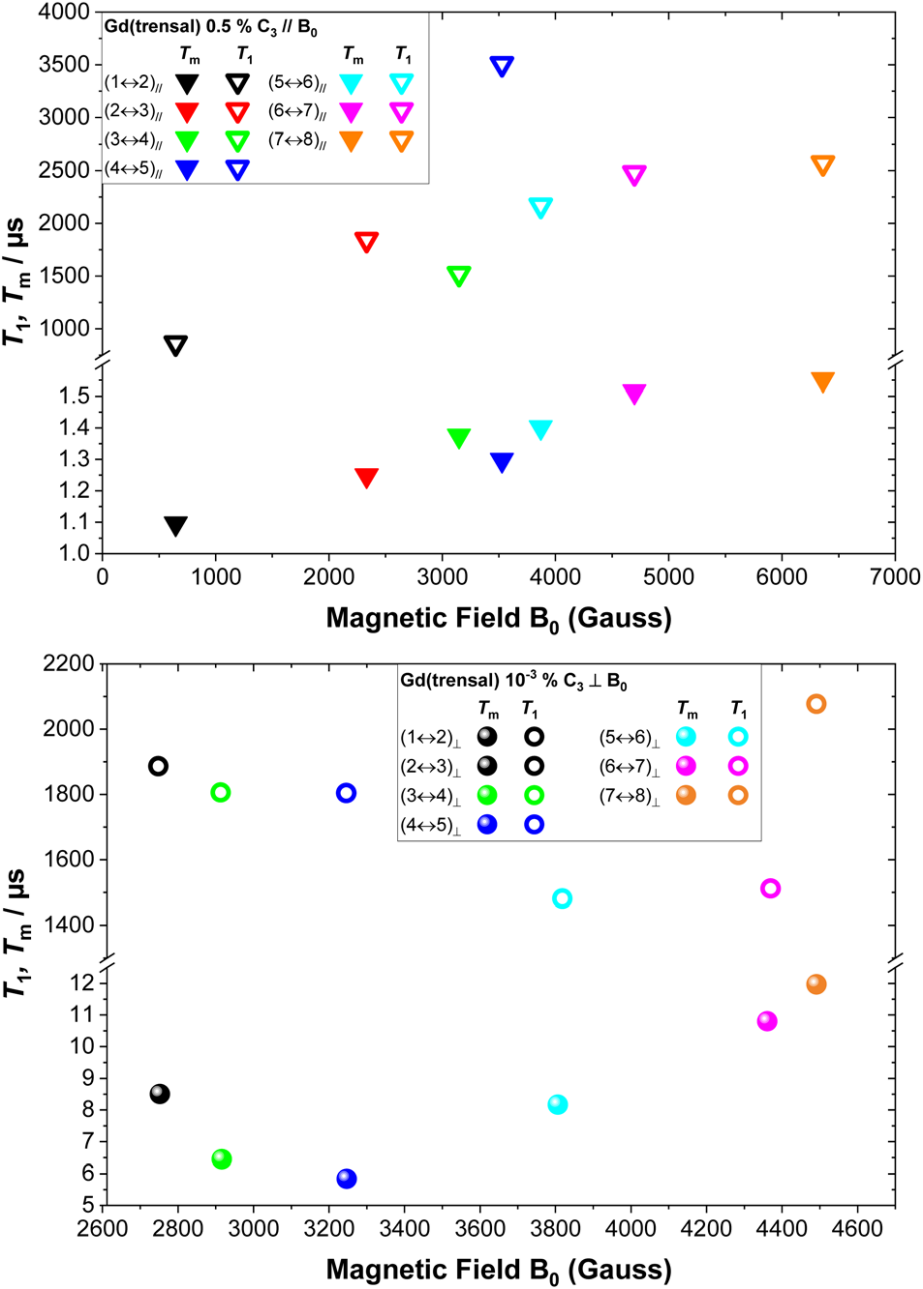
Field dependence of *T*_1_ and *T*_m_ for **0.5%** with *B*_0_ ∥ *C*_3_ (top) and for **10**^**−3**^**%** with *B*_0_ ⊥ *C*_3_ (bottom) at 5.5 K.

Longitudinal (*T*_1_) and transverse (*T*_m_) relaxation measurements were performed on each of the above-mentioned observed lines (Fig. S6–S32†), as described in previous sections. The measured *T*_1_ values for the various investigated EPR transitions are significantly different. For **0.5%** and *B*_0_ ∥ *C*_3_, an increase of *T*_1_ with magnetic field is observed (Fig. 3, top), where *T*_1_ increases from the lowest value of 863 μs for the (1 ↔ 2)_∥_ transition to the highest value at 3.5 ms for the (4 ↔ 5)_∥_ transition (Fig. 3, top), a fourfold increase. Qualitatively similar results are obtained for **0.5%** and *B*_0_ ⊥ *C*_3_ (Fig. S43†). In contrast, for **10**^**−3**^**%** and *B*_0_ ⊥ *C*_3_, the longest *T*_1_ is observed for transition (7 ↔ 8)_⊥_ with the (5 ↔ 6)_⊥_ transition having the shortest *T*_1_ (Fig. 3, bottom). This indicates that both the magnetic field and the nature of the eigenvectors involved in the particular EPR transition influence *T*_1_, both doing so *via* the magnetoelastic coupling terms 〈*f*|∂*Ĥ*/∂*R*|*i*〉, with |*i*〉 and |*f*〉 the eigenvectors involved in the transition and ∂*Ĥ*/∂*R* the derivative of the Hamiltonian due to the ∂*R* displacement.^70,71^ Furthermore, as previously discussed, a very large increase in *T*_1_ was observed going from high field/frequency measurements to lower field/frequency ones. For **0.5%** and *B*_0_ ∥ *C*_3_, the *T*_1_ of the (7 ↔ 8)_∥_ transition (Fig. 3, top) increases by two orders of magnitude with respect to the one determined at 240 GHz, from 24 μs at 240 GHz to 2.5 ms at 9.7 GHz at 5.5 K, hence, accordingly lifting the upper *T*_m_ limit. However, the observed *T*_m_ does not follow this increase in *T*_1_. In contrast, the (7 ↔ 8)_∥_ transition has a significantly lower *T*_m_ at X-band than at 240 GHz (1.6 μs *versus* 4.3 μs, respectively). As previously mentioned, the relative contributions to decoherence (Δ*ω*/*ω*, with Δ*ω* the dephasing) induced from local magnetic field fluctuations due to environmental dynamics decrease with increasing transition frequency, *ω*. This results in a progressive insensitivity of the dynamic state towards decoherence with increasing transition frequency. For **0.5%** and *B*_0_ ∥ *C*_3_ a slight increase of *T*_m_ with field is observed while for **10**^**−3**^**%** and *B*_0_ ⊥ *C*_3_, *T*_m_ shows non-monotonic behaviour with the central line (4 ↔ 5)_⊥_ transition (Fig. 3, bottom) displaying the shortest *T*_m_. Again, as for *T*_1_, both the magnetic field and the composition of the involved eigenstates influence *T*_m_. Qualitatively similar results are obtained for **0.5%** and *B*_0_ ⊥ *C*_3_ (Fig. S43†).

**Fig. 4:**
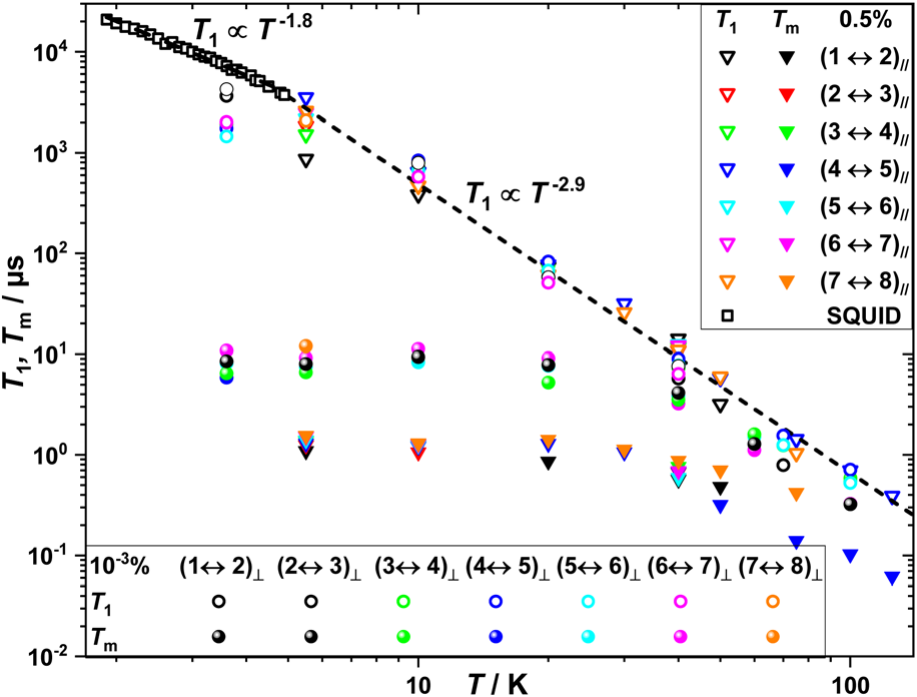
Temperature dependence of *T*_1_ and *T*_m_ obtained by EPR measurements of **0.5%** and **10**^**−3**^**%** with *B*_0_ ∥ *C*_3_ and *B*_0_ ⊥ *C*_3_. The dashed lines are linear fits to log(*T*_1_) *vs.* log(*T*) as described in the main text.

With respect to the temperature, *T*, dependence of the relaxation dynamics, as anticipated, *T*_1_ of all investigated resonances is highly temperature dependent, decreasing by three to four orders of magnitude from 5 to 125 K (Fig. 4). At X-band, a *T*_1_ ∝ *T*^−2.9^ dependence is observed in this *T* range, which is a higher exponent dependence than what observed at 240 GHz (*T*_1_ ∝ *T*^−0.44^) reflecting the increasing dominance with magnetic field of the direct process as compared to Raman ones. Dynamic susceptibility thermodynamic SQUID measurements of **0.5%** between 4.9 and 1.9 K (Fig. 4, S1, S2 and Tables S1, S2†) revealed a *T*_1_ ∝ *T*^−1.8^ dependence indicating that longitudinal relaxation is determined by a combination of processes, likely direct and Raman ones, as determined in previous studies.^65,80^*T*_1_ values obtained by pulse EPR measurements below 5 K deviate from the *T*_1_ ∝ *T*^−2.9^ behaviour and are closer to the *T*_1_ ∝ *T*^−1.8^ dependence.

### Sources of decoherence

Given that at low temperatures the determined *T*_m_ of Gd(trensal) at X-band is about two to three orders of magnitude lower than the corresponding *T*_1_ (Fig. 4), we now investigate the factors that limit it under these experimental conditions. In contrast to *T*_1_, *T*_m_ shows little temperature dependence up to about 40 K (Fig. 4). Above this temperature, *T*_m_ becomes limited by *T*_1_ and hence decreases accordingly, ultimately reaching the lowest detectable by our instrument level at about 100 K for *B*_0_ ⊥ *C*_3_ and 125 K for *B*_0_ ∥ *C*_3_.

**Fig. 5:**
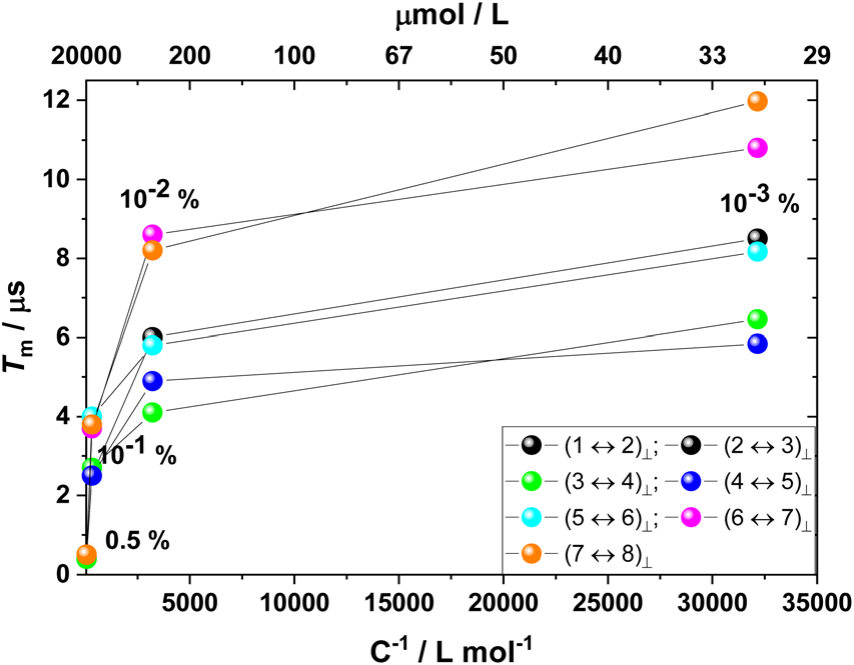
Inverse concentration dependence of *T*_m_ with *B*_0_ ⊥ *C*_3_ at 5 (**10**^**−1**^**%** and **10**^**−2**^**%**) and 5.5 (**0.5%** and **10**^**−3**^**%**) K. The solid lines are guides for the eye.

Magnetic dipole interactions between Gd(trensal) moieties distributed within the diamagnetic Y(trensal) lattice induce decoherence *via* dipolar magnetic field fluctuations due to indirect spin flip-flops involving neighbouring Gd(trensal) sites. The local magnetic field, *B*_loc_, generated by a magnetic dipole at distance *r* from the dipole is inversely proportional to the cube of *r* (*B*_loc_ ∝ *r*^−3^). Thus, magnetic field fluctuations due to dipolar interactions should also scale accordingly. Under these conditions, one would expect an inverse linear dependence of *T*_m_ to the concentration of paramagnetic sites in the host lattice until indirect electron–electron dipolar interactions cease to constitute the limiting factor. However, it has been shown that deviations from this simple behaviour can be observed when the coupling of the paramagnet to the spin bath, as well as the dynamics of the spin bath, are explicitly taken into account.^83^ To determine the dependence of *T*_m_ on the concentration, *C*, of Gd(trensal) sites in the Y(trensal) lattice, we studied samples diluted between 0.5 and 10^−3^% **(0.5%**, **10**^**−1**^**%**, **10**^**−2**^**%** and **10**^**−3**^**%**). For *B*_0_ ⊥ *C*_3_, we observe that while *T*_m_ initially increases fast with inverse concentration, at doping levels between **10^−2^%** and **10^−3^%***T*_m_ increases only marginally with *C*^−1^, thus shows signs of saturation (Fig. 5). This indicates that at the lowest studied doping level of **10^−3^%**, corresponding to a 30 μM molarity for Gd(trensal), dipolar interactions cease to be the dominating limiting factor for *T*_m_ (Fig. 5). Given that the typical volume of a herein measured Gd(trensal) crystal is of the order of 50 mm^3^, this corresponds to about 10^15^ measured spins at a doping level of **10^−3^%**, which corresponds to the lowest limit of number of spins that can be measured within a convenient amount of time (few minutes) with our experimental setup at X-band. Thus, while diluting from **0.5%** to **10**^**−3**^**%** led roughly to an increase of an order of magnitude in *T*_m_, further dilution is not expected to lead to a further substantial increase in *T*_m_. In addition, the paramagnetic ion content of samples more dilute than 10^−3^ reaches the detection limit of our setup at X-band, estimated to be roughly 10^12^ to 10^13^ spins, and would require much longer measurement times to achieve satisfactory signal to noise ratios.

**Fig. 6:**
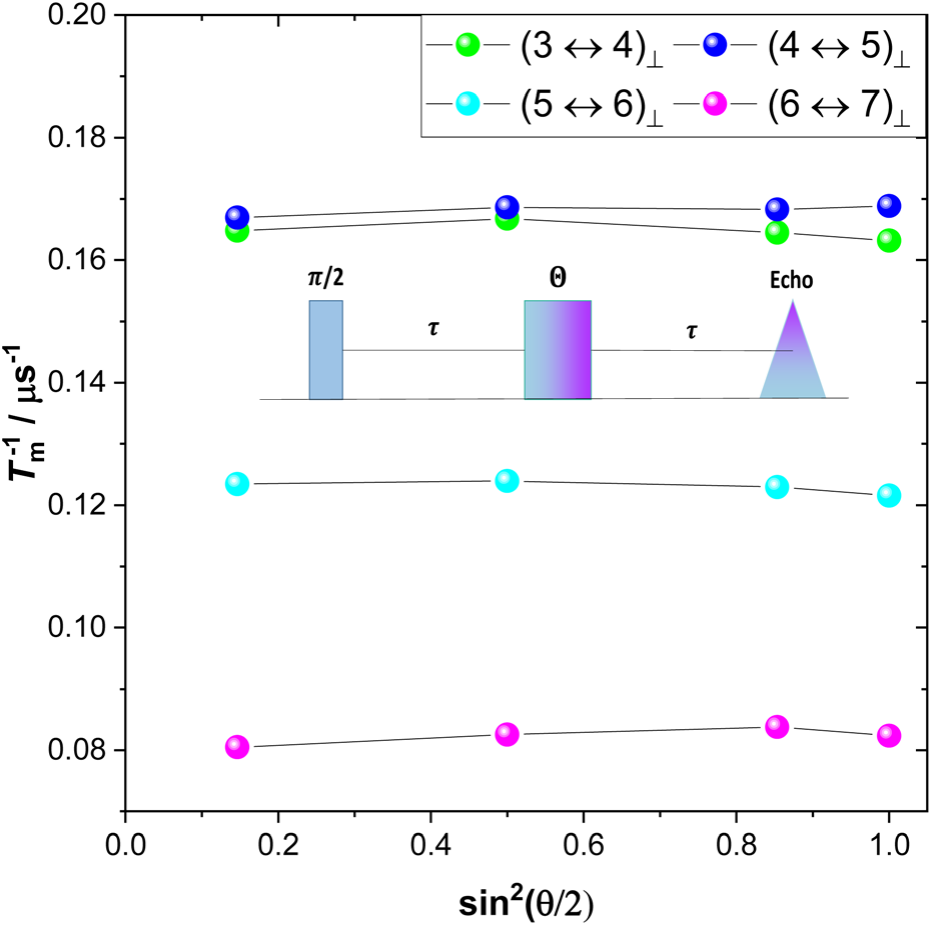
*T*_m_ of **10**^**−3**^**%** with *B*_0_ ⊥ *C*_3_ at 5.5 K measured *via* a Hahn echo sequence with variable length of the refocusing pulse at 4367, 3817, 3249 and 2917 Gauss, showing no angle dependence. The solid lines are guides for the eye.

Instantaneous diffusion contributions to decoherence occur as a consequence of neighbouring spins to the observed one being excited by the electromagnetic excitation pulse, hence causing a variation of the local field at the observed site. Instantaneous diffusion is often the limiting factor for impurities or defects in spin-free solid state materials such as silicon or diamond.^76,77^ The occurrence of this decoherence mechanism can be investigated by varying the macroscopic turning angle, *Θ*, of the refocusing pulses employed in a Hahn echo sequence, hence addressing fewer spins at the microscopic level. The dependence of *T*_m_ to *Θ* is expected to follow:^67,76^
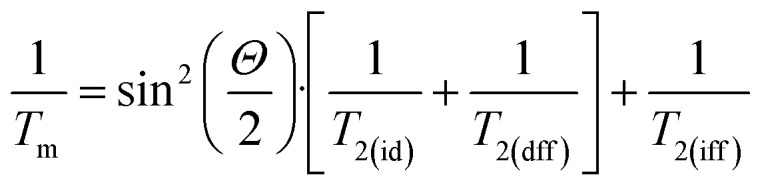
with *T*_2(id)_, *T*_2(dff)_ and *T*_2(iff)_ being the instantaneous diffusion, direct flip flop and indirect flip flop contributions to *T*_m_, respectively. *T*_2(dff)_ is often small compared to the other terms. Both *T*_2(id)_ and *T*_2(dff)_ scale as sin^2^(*Θ*/2), while *T*_2(iff)_ is independent of *Θ*. Thus, extrapolation to *Θ* = 0 corresponds to a decoherence regime where complete suppression of the instantaneous diffusion and direct flip flop contributions occurs, leaving only the one from indirect flip flops (*T*^−1^_2(iff)_) which is also called spectral diffusion. The experimental realisation of this regime is achieved by either reducing the amplitude of the magnetic field component (*B*_1_), either the length, of the refocusing pulse (*Θ* pulse in Fig. 6) with respect to what required for a π pulse. Herein we investigate resolved resonances from single crystals probing unique molecular orientations. Thus, we chose to vary the length of the refocusing pulse as the change in the excitation profile related to the variation of the pulse length is not expected to result in different orientational sub-populations being probed. Negligible variation of *T*_m_ with *Θ* was observed for all the investigated transitions (Fig. 6 and S33–S36†) suggesting that direct flip-flops and instantaneous diffusion are not dominant decoherence mechanisms under these experimental conditions. This is also expected as the sample is relatively highly magnetically dilute. Furthermore, the thermal population distribution of the m_*S*_ sublevels of Gd(iii) results in only a single sublevel being resonantly addressed out of 8 possible, effectively resulting in a smaller fraction of the spins being in resonance with the microwave pulse.

**Fig. 7:**
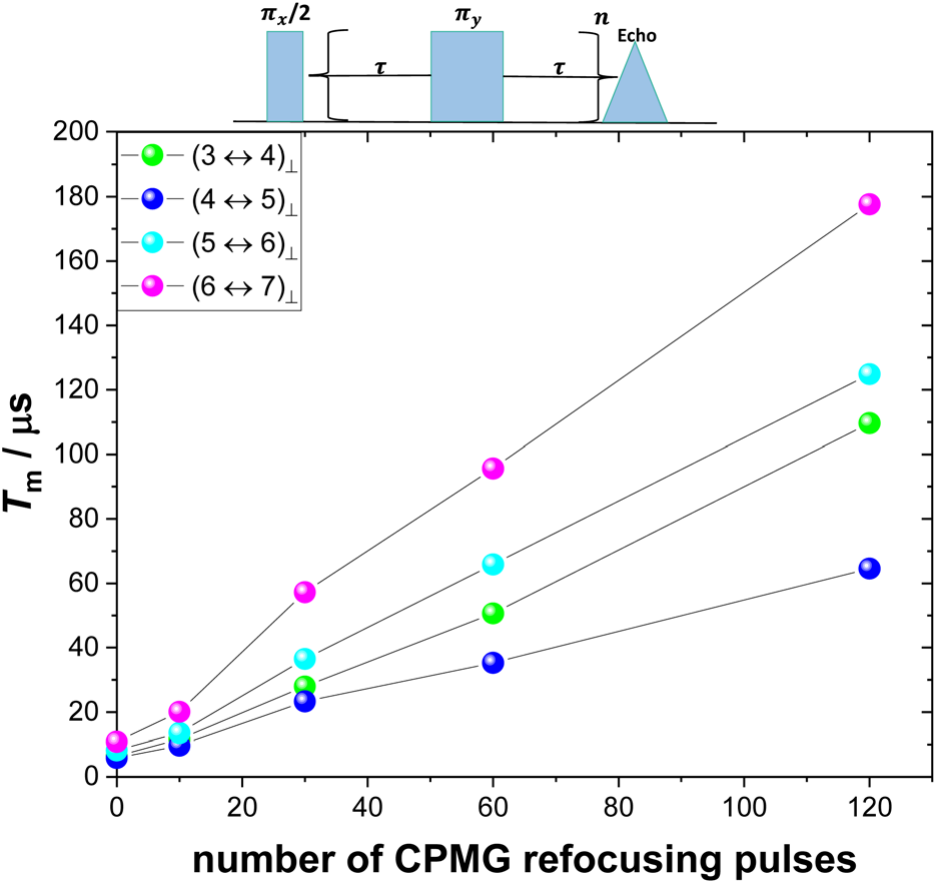
*T*_m_ of **10**^**−3**^**%** with *B*_0_ ⊥ *C*_3_ at 5.5 K as a function of the number of refocusing CPMG pulses at 4367, 3817, 3249 and 2917 Gauss. The solid lines are guides for the eye.

Decoherence originating from indirect flip flops (spectral diffusion) occurs as a consequence of the stochastic noise of nearby spin pairs flipping during the Hahn echo sequence. Spectral diffusion effects can be remediated for by dynamic decoupling,^67,84,85^ for example by employing the CPMG pulse sequence where a π-pulse-train refocuses the transverse magnetization.^67,78,79^ This effectively acts as a frequency filter for magnetic noise, giving rise to much longer transverse relaxation time. Application of the CPMG pulse sequence for **10**^**−3**^**%** with *B*_0_ ⊥ *C*_3_ and varying length of the pulse train revealed a substantial increase of *T*_m_ (Fig. 7 and S37–S39 and Table S6†). In particular, for the (6 ↔ 7)_⊥_ transition at 4367 Gauss, *T*_m_ increases from about 10.8 μs for *n* = 0 (Hahn echo) to around 180 μs for *n* = 180, thus by a factor of 16.4. The linear dependence of *T*_m_ on the length of the pulse train indicates that the dominant decoherence mechanism under these experimental conditions is spectral diffusion, which is also expected given the large number of nuclear spin bearing atoms present in the studied crystals. In particular, hydrogen atoms of the lattice are expected to heavily induce decoherence.^41^ Thus, we show herein that repetitive refocusing of the transverse magnetisation can substantially increase *T*_m_ in nuclear spin-rich media, by dynamically suppressing nuclear spectral diffusion.

**Fig. 8:**
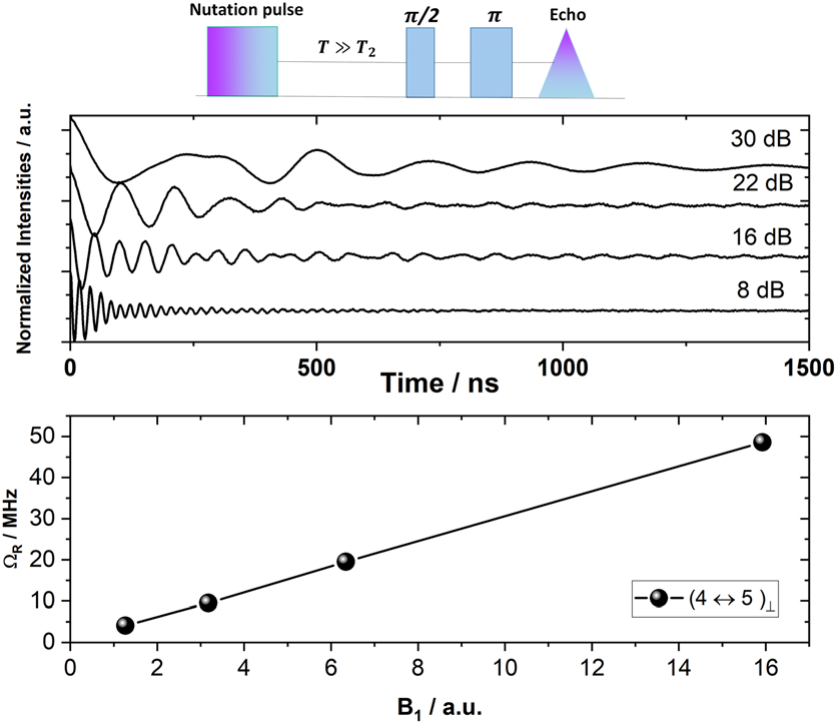
(Top): Schematic of the transient nutation pulse sequence and associated Rabi oscillations of **10**^**−3**^**%** with *B*_0_ ⊥ *C*_3_ at 5.5 K for the central (4 ↔ 5)_⊥_ transition. (Bottom): Rabi frequencies plotted against the relative magnitude of the magnetic field component of the microwave pulse (B1).

The multilevel structure of molecular magnetic materials has been proposed for the implementation of quantum error correction algorithms or of multiple qubits. For this purpose, the ability to place in a coherent superposition any two arbitrary eigenstates, say |***i***〉 and |***j***〉, of the system is required. However, it has been shown that this universality can also be achieved by sequentially addressing several allowed transitions connecting the two eigenstates of interest, |***i***〉 and |***j***〉, with other eigenstates of the Hilbert space, say |***k***〉. Thus a coherent superposition of |***i***〉 and |***j***〉 can be achieved *via* the |***k***〉 states (|***i***〉↔|***k***〉↔|***j***〉).^86^ To demonstrate that the spin eigenstates of Gd(trensal) can be placed in coherent superposition, we performed transient nutation experiments (*θ*–*T*–π/2–*τ*–π–*τ*–echo, Fig. 8 and S40–S42†). Using a Fourier transform of the time trace of the echo evolution, the Rabi frequency, *Ω*_R_, at each power level for the different allowed transitions was obtained. For Rabi oscillations, *Ω*_R_ scales linearly with microwave power, as:
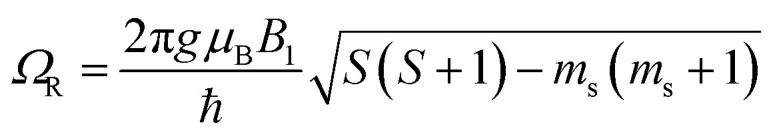
with *g* the *g*-factor, *μ*_B_ the Bohr magneton, *B*_1_ the magnetic field of the microwave pulse, ℏ the reduced Planck constant and *S* and m_S_ the spin angular momentum and its projection on the quantisation axis, respectively. A linear fit to the Rabi oscillation frequency as a function of *B*_1_ shows that the oscillations observed in the nutation experiment are indeed Rabi oscillations (Fig. 8 and S42†).

The above described Rabi-nutation experiments also provide the timescale, *T*_G_, for single qubit gates, for example π or π/2 rotations. It can for example be seen from Fig. 8, that the duration of a π rotation is of the order of 10 ns at the highest power investigated (9 dB). This time duration for a π gate combined with the long coherence times achieved *via* CPMG, results in a *T*_m_/*T*_G_ ratio of the order of 10^4^ (Table 1), which is the desired order of magnitude for the figure of merit of a qubit. This demonstrates that quantum gates implemented on molecular materials can reach fidelities of 99.99% or above.

**Table 1:** π-Rotation gate durations, *T*_G_, associated phase memory times, *T*_m_ and *T*_m_/*T*_g_ ratios for four different allowed transitions of Gd(trensal) **10^−3^%** with *B*_0_ ⊥ *C*_3_

Transition	*T*_G_ (π-rotation)/ns	*T*_m_/μs	*T*_m_/*T*_G_
(3 ↔ 4)_⊥_	12	109	9083
(4 ↔ 5)_⊥_	12	64	5333
(5 ↔ 6)_⊥_	10	124	12 400
(6 ↔ 7)_⊥_	12	178	14 833

## Conclusions

We undertook herein a systematic investigation of the mechanisms that induce decoherence in qubits implemented in molecular magnetic materials. We show that in the case that these processes are probed by pulse EPR under the most usual experimental conditions (X-band), nuclear spin spectral diffusion is the dominant process determining decoherence. However, spectral diffusion effects can be remediated for by dynamic refocusing sequences, such as CPMG, even in such dense nuclear spin environments. For the materials investigated herein, the bulk concentration is of 3 M which, for example, corresponds to a concentration of proton nuclear spins of the order of 80 M, given that each molecule has 27 protons. This is a relatively large concentration of nuclear spins that dynamic refocusing is able to remediate for. Thus, it is not absolutely necessary to utilise nuclear spin free environments, such as frozen solution in solvents devoid of nuclear spins, to study the coherent properties of these materials. Furthermore, use of dynamic decoupling allowed *T*_m_ of the investigated materials to be increased by two orders of magnitude. This resulted in reaching 180 μs for 120 refocusing pulses with the increase in *T*_m_ being linear to the number of refocusing pulses, thus without showing signs of saturation. The obtained *T*_m_'s combined with *T*_G_'s of the order of 10 ns leads to qubit figure of merit ratios of the order of 10^4^. This demonstrates the suitability of molecular magnetic materials for use as hardware for quantum technologies. Chemical engineering of organic scaffolds where protons are exchanged for deuterium that has a significantly lower magnetic moment than protons would allow use of pulse sequences not including dynamic refocusing. Detection of an echo at relatively high temperatures (125 K) indicates that further tuning of the dynamic magnetic properties of lanthanide-based molecular materials can lead to coherent magnetic properties at even higher temperatures, as has been demonstrated in the case of transition metal complexes. Finally, we provide herein detailed solid state relaxation data of lanthanide coordination complexes, obtained on magnetically dilute single crystals of trigonal symmetry.

## Data availability

Data for this article, including pulsed EPR data are available as ESI.†

## Author contributions

The project was jointly conceived by CDB, SHH and SP. CDB and AS prepared the samples. JBP, MUIC, MR and SHH performed the measurements. CDB, JBP and SHH performed the data analysis, supervised by SP, REPW and EJLM. The manuscript was written jointly by all authors, who all have read and agreed to the final version of the manuscript.

## Conflicts of interest

There are no conflicts to declare.

## Supplementary Material

SC-OLF-D4SC05304D-s001

SC-OLF-D4SC05304D-s002
