# Supplementary material for: Probing decoherence in molecular 4f qubits
Source: Chem Sci. 2024 Oct 30;15(48):20328–37. doi: 10.1039/d4sc05304d (PMC11575486; doi:10.1039/d4sc05304d)
Supplement: SC-015-D4SC05304D-s002 [file SC-015-D4SC05304D-s002.pdf]

Supplementary Information (SI) for Chemical Science.
This journal is © The Royal Society of Chemistry 2024

Probing Decoherence in Molecular 4f Qubits

Steen H. Hansen,[†,1] Christian D. Buch,[†,1] Jonatan B. Petersen,[2] Michelle Rix,[1] Marc Ubach I Cervera,[1] Asger Strandfelt,[1] Richard E. P. Winpenny,[2] Eric J. L. McInnes,[2] and Stergios Piligkos[1,*]

[1] University of Copenhagen, Universitetsparken 5, DK-2100 Copenhagen, Denmark

[2] Department of Chemistry, School of Natural Science, The University of Manchester, Oxford Road, Manchester M13 9PL, United Kingdom

Contents

SQUID magnetometry S2

EPR spectroscopy S5

Hamiltonian parameters and energy spectrum S6

EPR relaxation measurements S7

CPMG S26

Rabi oscillations S29

SQUID magnetometry

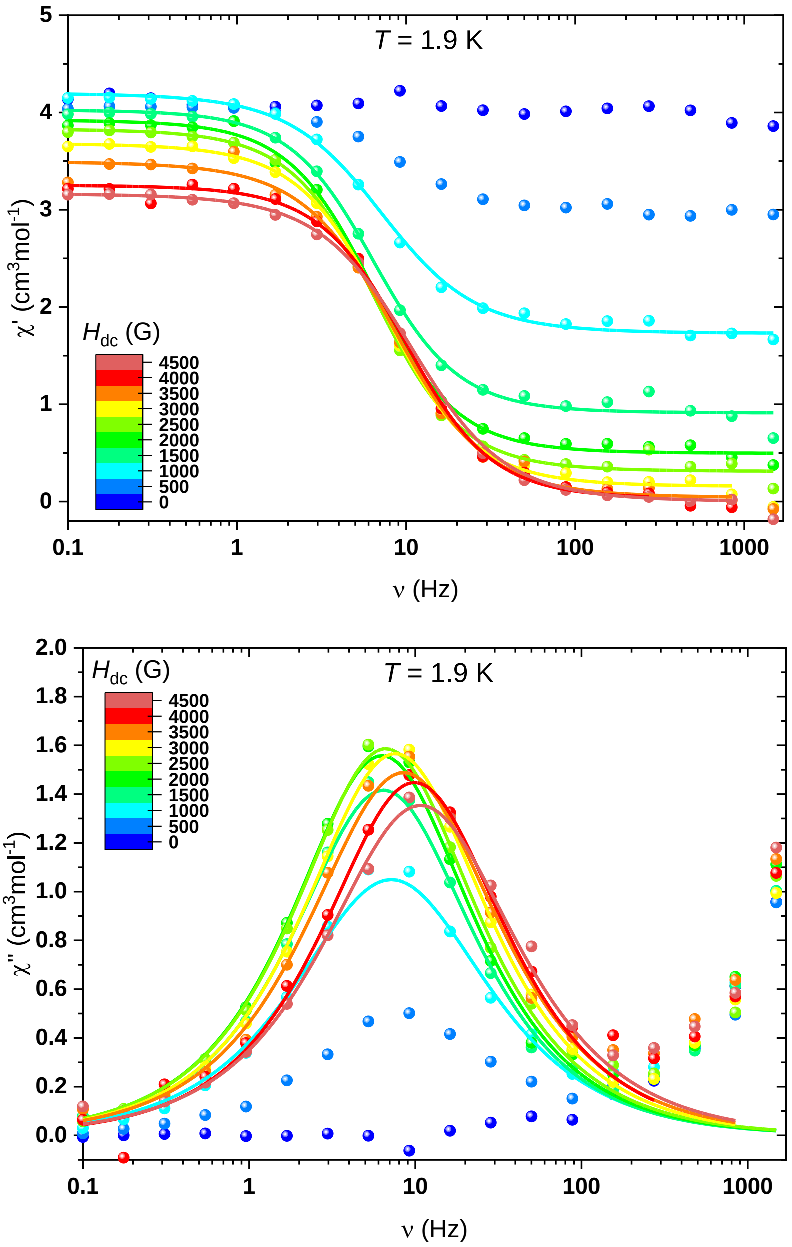

Figure S1. Field dependence of the in-phase (top) and out-of-phase (bottom) AC susceptibility of a single crystal of $Gd_{0.05}Y_{0.95}$(trensal) (**0.5%**) placed with the molecular C_3 axis parallel to the external magnetic field ($B_0 \| C_3$). Experiment is in scatter and the best-fit to a generalized Debye model as explained in the main text is is shown as coloured lines.

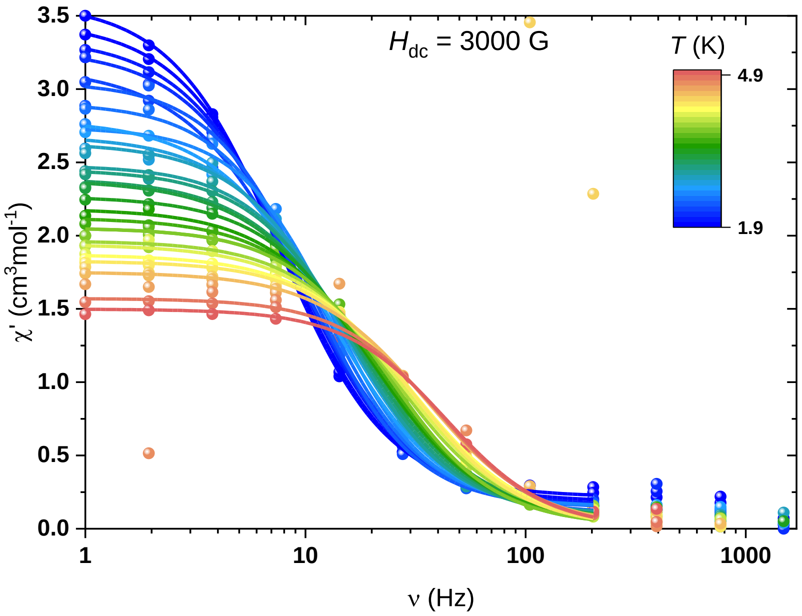

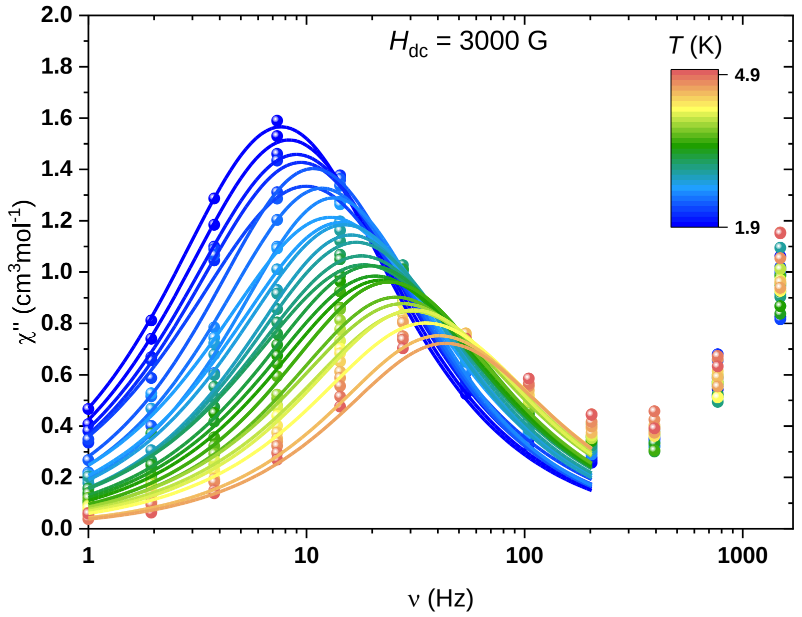

Figure S2. Temperature dependence of the in-phase (top) and out-of-phase (bottom) AC susceptibility of a single crystal of $Gd_{0.05}Y_{0.95}$(trensal) (**0.5%**) placed with the molecular C_3 axis parallel to the external magnetic field ($B_0 \| C_3$) of 3000 G. Experiment is in scatter and the best-fit to a generalized Debye model as explained in the main text is is shown as coloured lines.

Table S1. Values of τ and α including errors for a single crystal of $Gd_{0.05}Y_{0.95}$(trensal) with $B_o \| C_3$ at 1.9 K and varying magnetic field. The data were extracted from a fit of the ac magnetic susceptibility to a generalized Debye model as described in the main text. The fits are shown in Figure S1.

B (G)	τ (ms)	τ error	α	α error
1000	22.2	2.9	0.103	0.070
1500	24.8	2.4	0.061	0.055
2000	25.3	2.4	0.061	0.053
2500	24.0	2.1	0.066	0.048
3000	21.0	1.0	0.075	0.026
3500	18.7	1.2	0.095	0.035
4000	16.1	0.5	0.069	0.019
4500	14.8	0.9	0.099	0.032

Table S2. Values of τ and α including errors for a single crystal of $Gd_{0.05}Y_{0.95}$(trensal) with $B_o \| C_3$ at at B_o = 3000 G and varying temperature. The data were extracted from a fit of the ac magnetic susceptibility to a generalized Debye model as described in the main text. The fits are shown in Figure S2.

T (K)	τ (ms)	τ error	α	α error
1.9	20.8	0.43	0.051	0.013
2.0	19.1	0.37	0.052	0.012
2.1	17.7	0.36	0.061	0.013
2.2	16.9	0.45	0.062	0.017
2.3	16.0	0.72	0.090	0.027
2.4	14.8	0.62	0.023	0.028
2.5	13.5	0.58	0.027	0.028
2.6	12.0	0.53	0.002	0.030
2.7	12.4	0.41	0.075	0.021
2.8	11.1	0.39	0.058	0.023
2.9	10.7	0.32	0.046	0.019
3.0	10.0	0.37	0.027	0.024
3.1	9.4	0.23	0.044	0.015
3.2	8.9	0.28	0.065	0.020
3.3	8.7	0.33	0.084	0.023
3.4	8.1	0.20	0.055	0.016
3.5	7.7	0.26	0.055	0.021
3.6	7.2	0.22	0.051	0.019
3.7	6.6	0.21	0.038	0.020
3.8	6.6	0.28	0.038	0.027
3.9	6.2	0.21	0.040	0.021
4.1	5.8	0.19	0.063	0.021
4.2	5.2	0.20	0.063	0.024
4.3	5.1	0.16	0.044	0.020
4.5	4.5	0.12	0.056	0.017
4.8	3.9	0.17	0.028	0.029
4.9	3.7	0.19	0.023	0.033

EPR spectroscopy

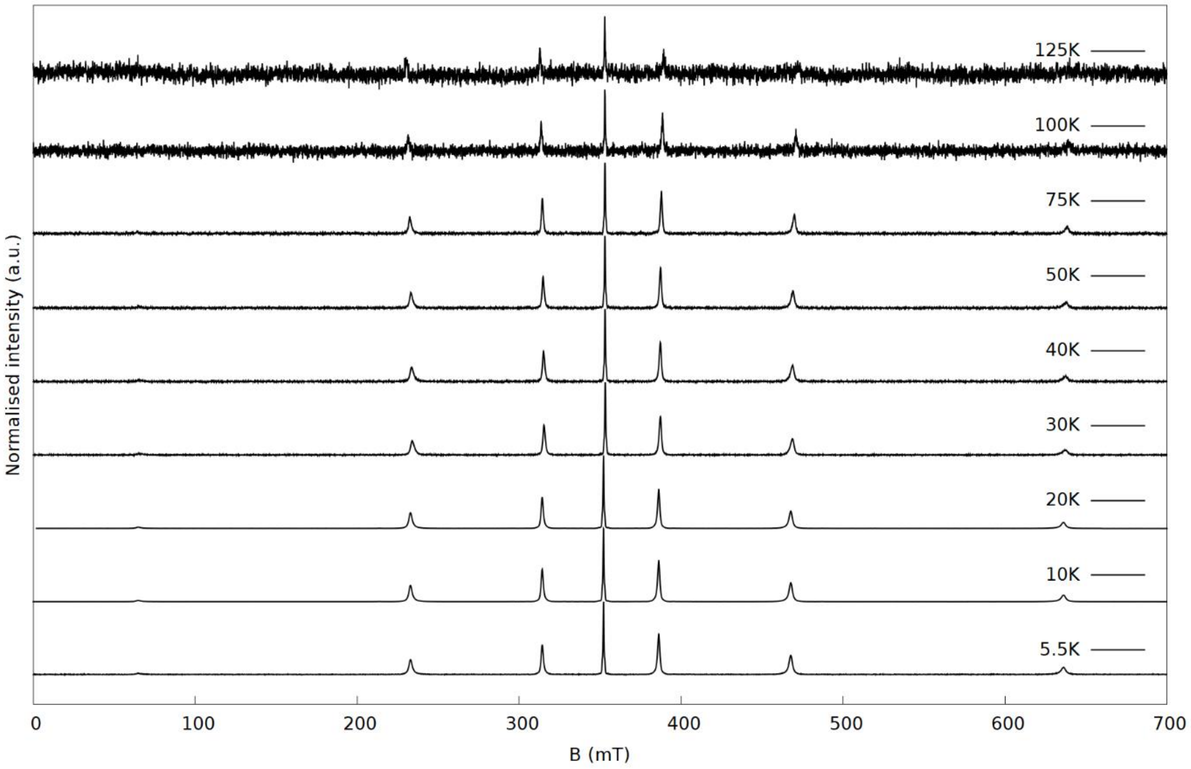

Figure S3. Echo Detected Field sweep spectra of **0.5%** with $B_0 \| C_3$ at various temperatures. At 100 K and 125 K the delay between the pulses in the echo sequence was set to τ=200 ns, to increase the echo intensity

Hamiltonian parameters and energy spectrum

The spectrum was simulated with the following Hamiltonian:

$$\hat{H} = \mu_B B g_{\|} \hat{s}_z + \sum_{k,q} B_k^q \hat{O}_k^q$$

Where the first term describes the Zeeman interaction, μ_B is the Bohr magneton, B is the field, $g_{\|}$ is the g-value along the unique axis and $\hat{s}_z$ is a spin operator. The second term describes the Zero Field Splitting of the ground state with Stevens operator equivalents $\hat{O}_k^q$ and Stevens parameters B_k^q.

Table S3. Parameters used for simulation of EDFS and Zeeman diagram.[1]

$g_{\|}$ & $g_{\perp}$	B_2^0 10^{-2} cm^{-1}	B_4^0 10^{-5} cm^{-1}	B_6^0 10^{-9} cm^{-1}	B_4^3 10^{-4} cm^{-1}	B_4^{-3} 10^{-4} cm^{-1}
1.992 & 1.985	1.14	4.84	-6.07	5.79	5.57

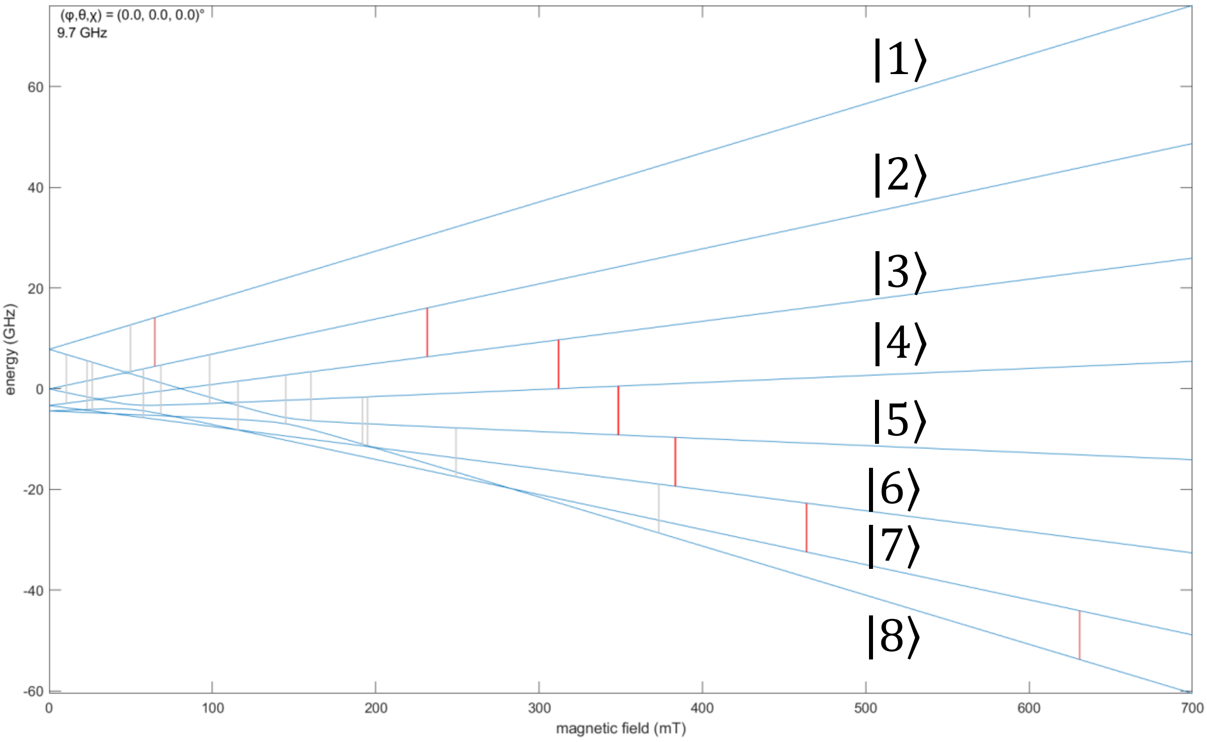

Figure S4. Simulation of the Zeeman splitting diagram of Gd(tremsal) with C_3 along the magnetic field calculated with the spin Hamiltonian parameters previously published. Red lines indicate allowed transitions and grey lines indicate forbidden transitions.

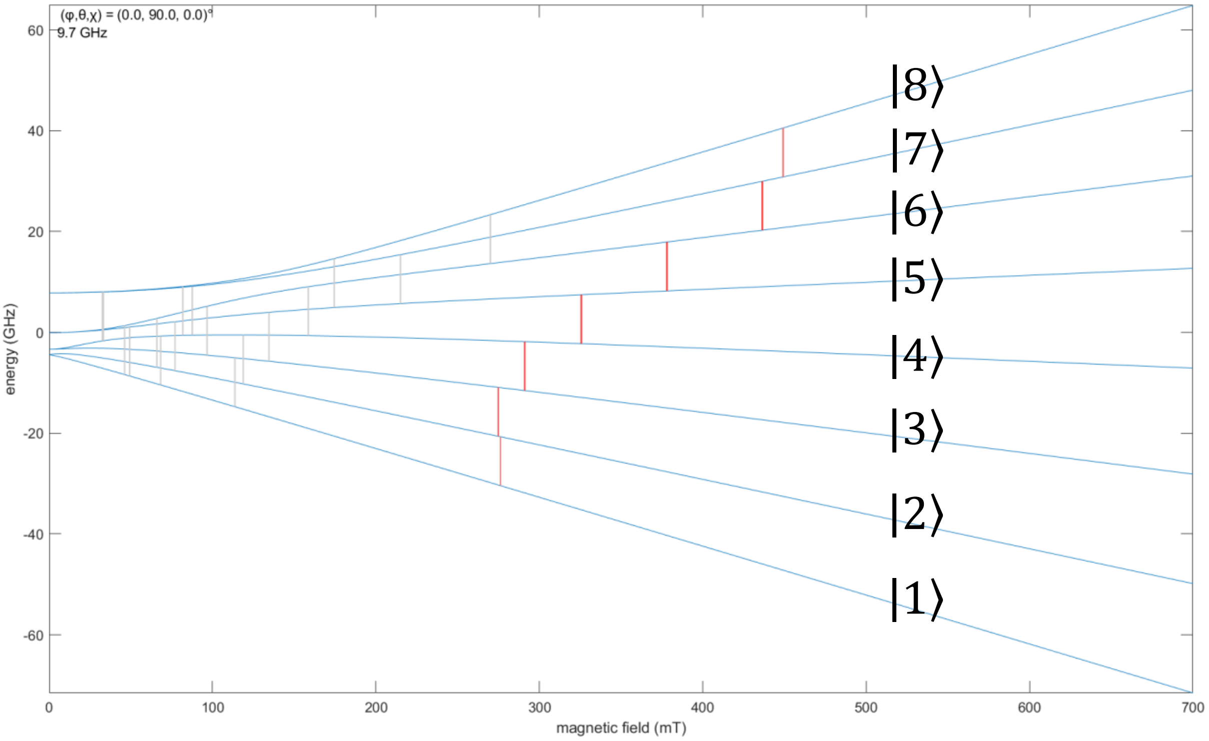

Figure S5. Simulation of the Zeeman splitting diagram of Gd(tremsal) with C_3 perpendicular to the magnetic field calculated with the spin Hamiltonian parameters previously published. Red lines indicate allowed transitions and grey lines indicate forbidden transitions.

EPR relaxation measurements

EPR relaxation data was fitted to the following mono- and bi-exponential functions:

$$I = I_0 + k \cdot e^{-\left(\frac{\tau_1}{T_1}\right)}$$

$$I = I_0 + k_1 \cdot e^{-\left(\frac{\tau_1}{T_{1\_1}}\right)} + k_2 \cdot e^{-\left(\frac{\tau_1}{T_{1\_2}}\right)}$$

for T_1 and

$$I = I_0 + k \cdot e^{-\left(\frac{2\tau}{T_m}\right)}$$

for T_m

Where I_0 is the y-offset, k is a proportionality constant, τ_1 is the time between the first pulse and the Hahn echo detection sequence within the inversion recovery sequence, τ is the time between pulses in the echo decay sequence.

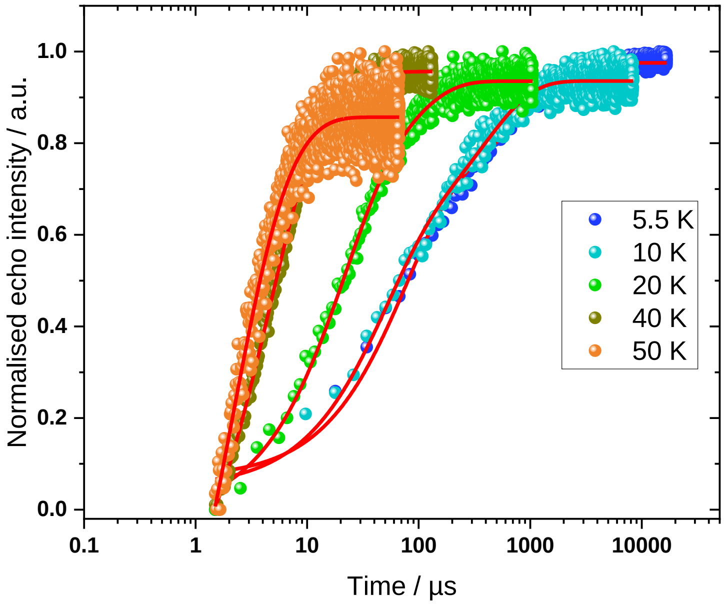

Figure S6. Normalised echo intensities of an inversion recovery sequence (scatter) measured for **0.5%** with $B_0 \| C_3$ at the $(7 \leftrightarrow 8)_{\|}$ transition at different temperatures. The best fit to a bi-exponential for 5.5 – 50 K is shown as red lines.

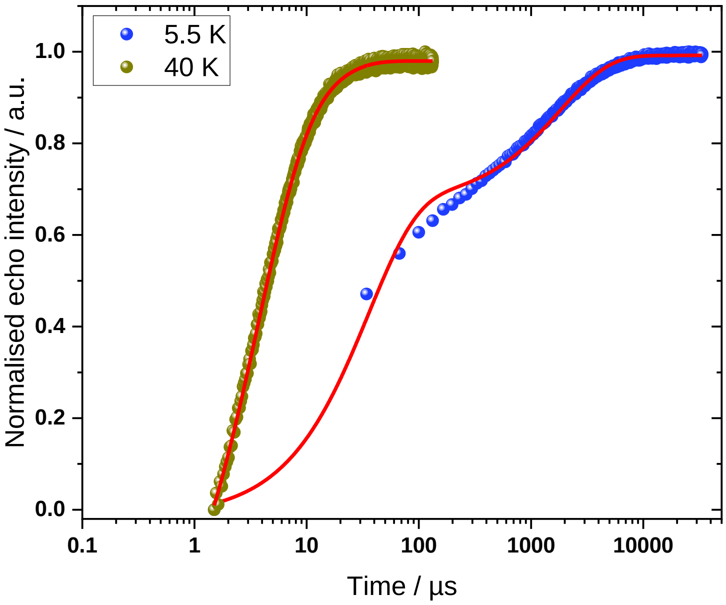

Figure S7. Normalised echo intensities of an inversion recovery sequence (scatter) measured for **0.5%** with $B_0 \| C_3$ at the $(6 \leftrightarrow 7)_{\|}$ transition at different temperatures. The best fit to a bi-exponential is shown as red lines.

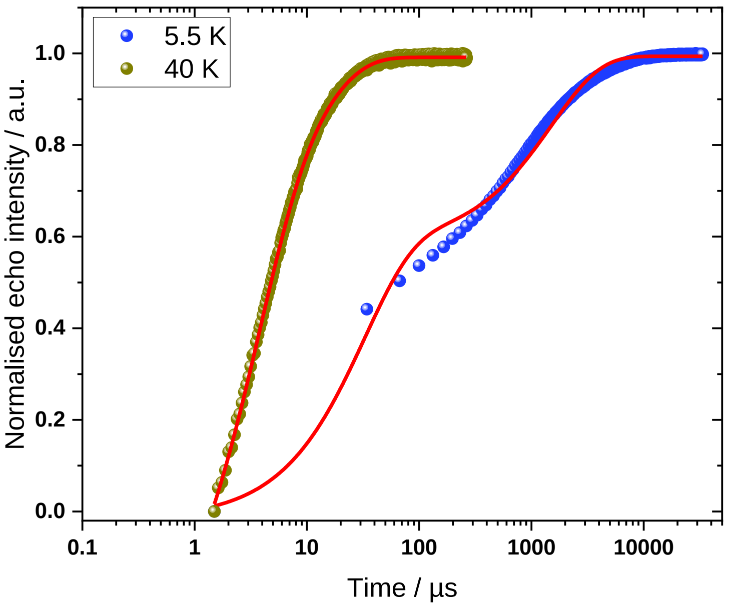

Figure S8. Normalised echo intensities of an inversion recovery sequence (scatter) measured for **0.5%** with $B_0 \| C_3$ at the $(5 \leftrightarrow 6)_{\|}$ transition at different temperatures. The best fit to a bi-exponential is shown as red lines.

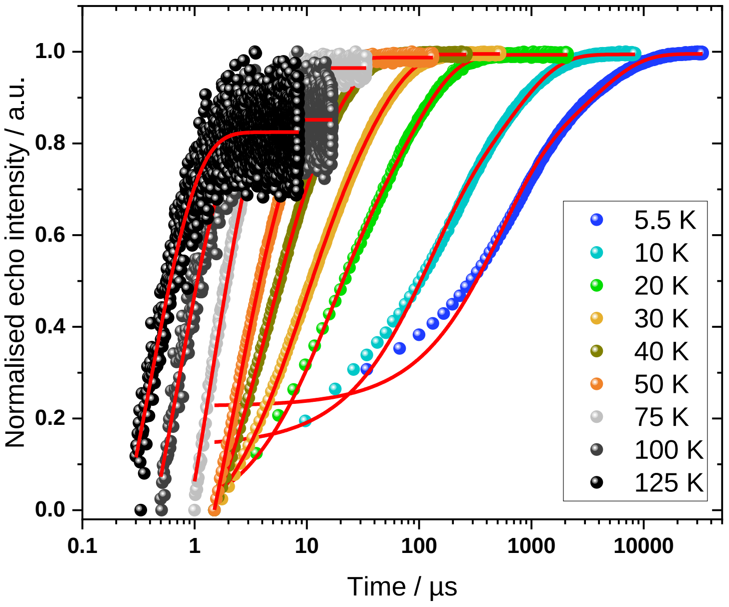

Figure S9. Normalised echo intensities of an inversion recovery sequence (scatter) measured for **0.5%** with $B_0 \| C_3$ at the $(4 \leftrightarrow 5)_{\|}$ transition at different temperatures. The best fit to a bi-exponential for 5.5 – 50 K and a monoexponential for 75 – 125 K is shown as red lines.

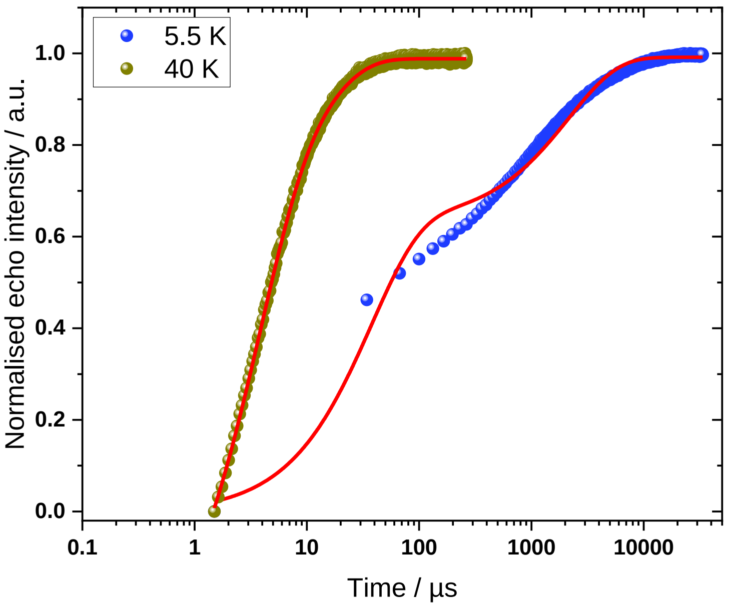

Figure S10. Normalised echo intensities of an inversion recovery sequence (scatter) measured for **0.5%** with $B_0 \| C_3$ at the $(3 \leftrightarrow 4)_{\|}$ transition at different temperatures. The best fit to a bi-exponential is shown as red lines.

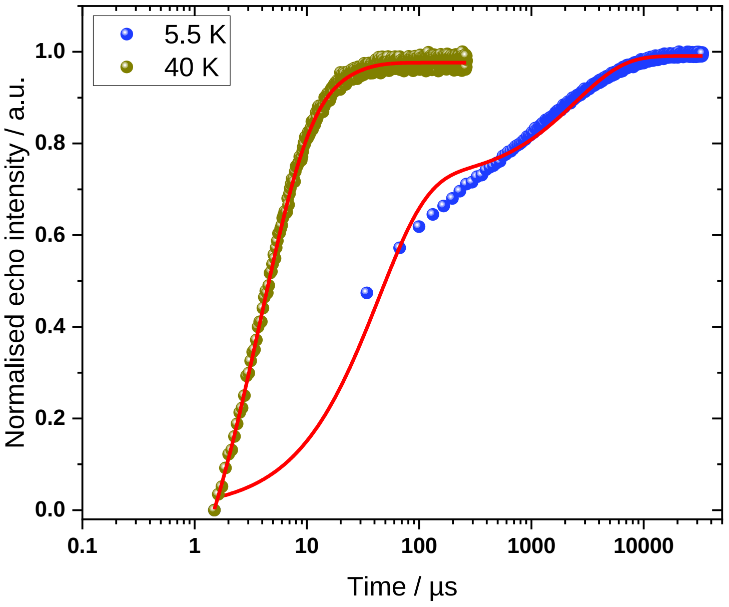

Figure S11. Normalised echo intensities of an inversion recovery sequence (scatter) measured for **0.5%** with $B_0 \| C_3$ at the $(2 \leftrightarrow 3)_{\|}$ transition at different temperatures. The best fit to a bi-exponential is shown as red lines.

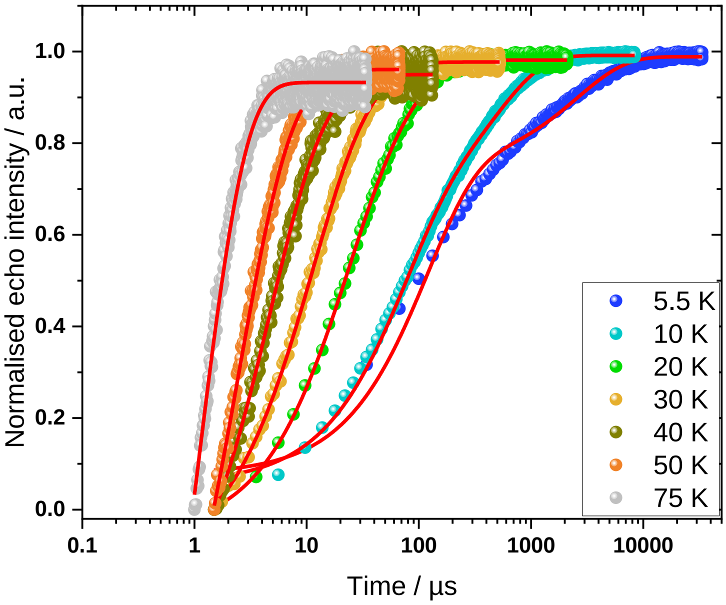

Figure S12. Normalised echo intensities of an inversion recovery sequence (scatter) measured for **0.5%** with $B_0 \| C_3$ at the $(1 \leftrightarrow 2)_{\|}$ transition at different temperatures. The best fit to a bi-exponential for 5.5 – 50 K and a monoexponential for 75 K is shown as red lines.

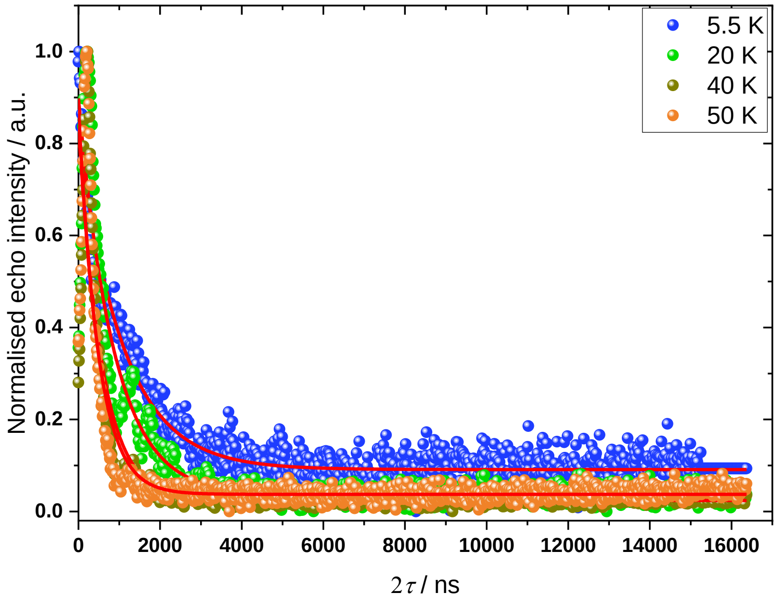

Figure S13. Normalised Hahn echo intensities (scatter) as a function of 2τ measured for **0.5%** with $B_0 \| C_3$ at the (7↔8)$_{\|}$ transition at different temperatures. The best fit to a mono exponential is shown as red lines.

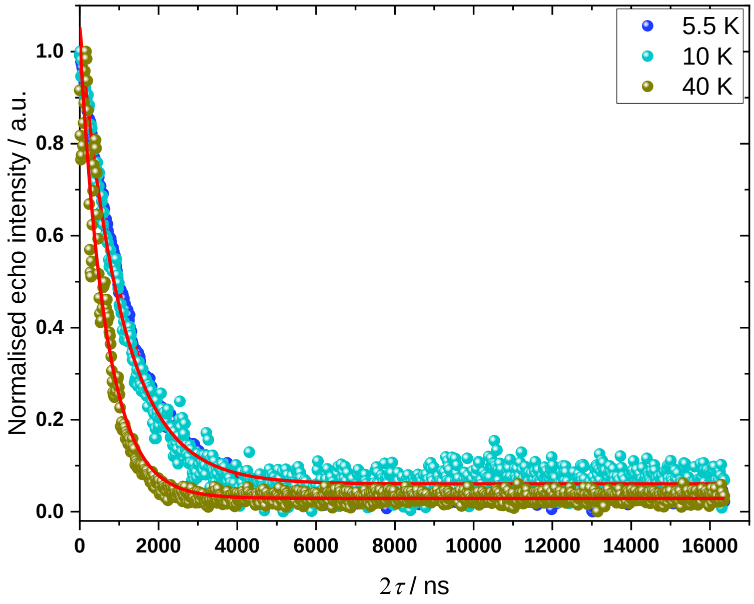

Figure S14. Normalised Hahn echo intensities (scatter) as a function of 2τ measured for **0.5%** with $B_0 \| C_3$ at the (6↔7)$_{\|}$ transition at different temperatures. The best fit to a mono exponential is shown as red lines.

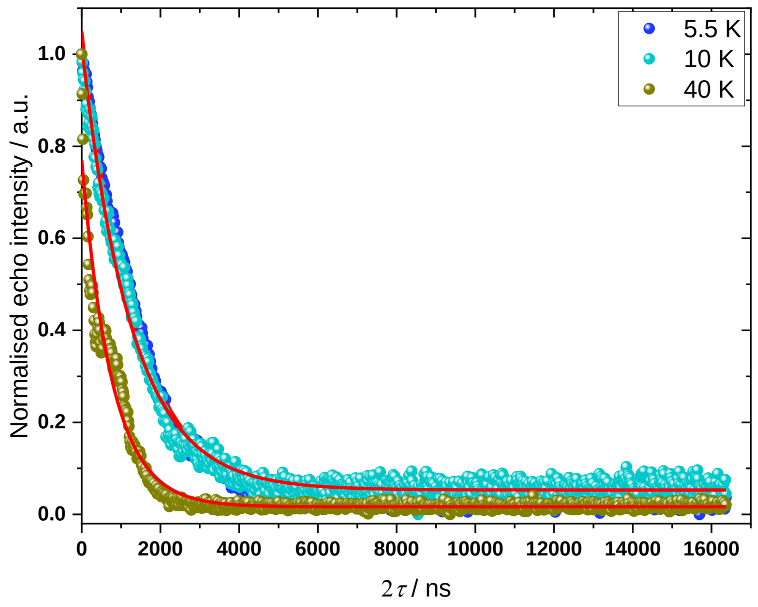

Figure S15. Normalised Hahn echo intensities (scatter) as a function of 2τ measured for **0.5%** with $B_0 \| C_3$ at the $(5\leftrightarrow6)_{\|}$ transition at different temperatures. The best fit to a mono exponential is shown as red lines.

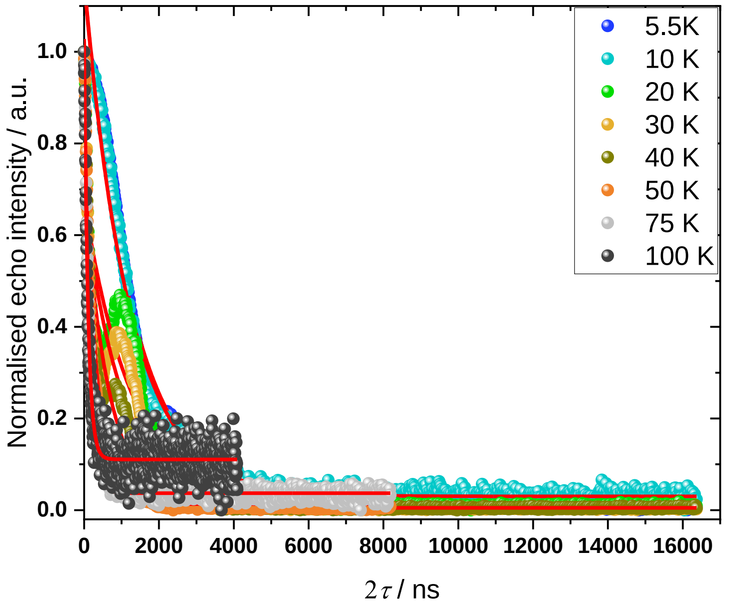

Figure S16. Normalised Hahn echo intensities (scatter) as a function of 2τ measured for **0.5%** with $B_0 \| C_3$ at the $(4\leftrightarrow5)_{\|}$ transition at different temperatures. The best fit to a mono exponential is shown as red lines.

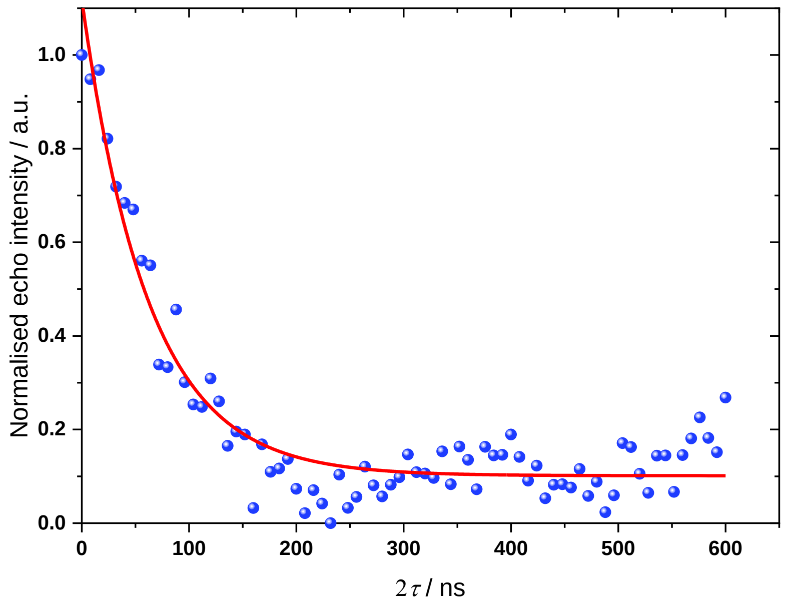

Figure S17. Normalised Hahn echo intensities (scatter) as a function of 2τ measured for **0.5%** with $B_0 \parallel C_3$ at the $(4\leftrightarrow5)_{\parallel}$ transition at 125 K. The best fit to a mono exponential is shown as red lines.

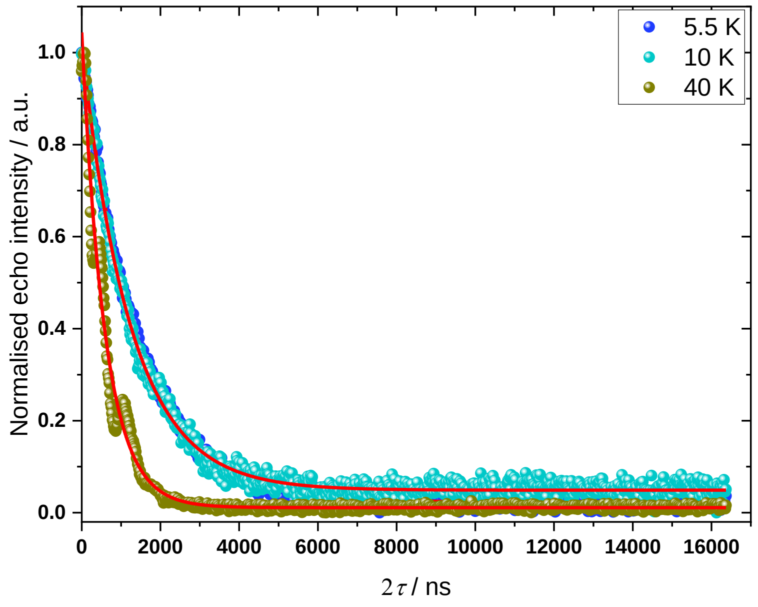

Figure S18. Normalised Hahn echo intensities (scatter) as a function of 2τ measured for **0.5%** with $B_0 \parallel C_3$ at the $(3\leftrightarrow4)_{\parallel}$ transition at different temperatures. The best fit to a mono exponential is shown as red lines.

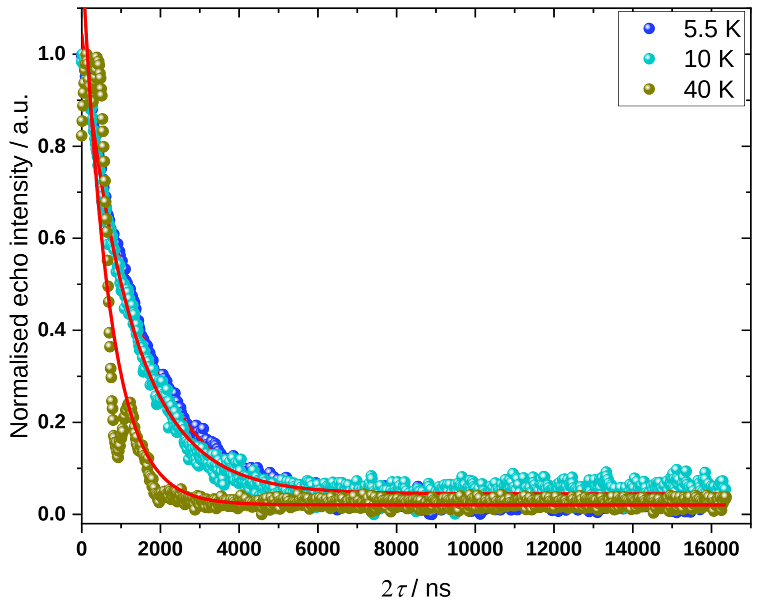

Figure S19. Normalised Hahn echo intensities (scatter) as a function of 2τ measured for **0.5%** with $B_0 \| C_3$ at the $(2 \leftrightarrow 3)_{\|}$ transition at different temperatures. The best fit to a mono exponential is shown as red lines.

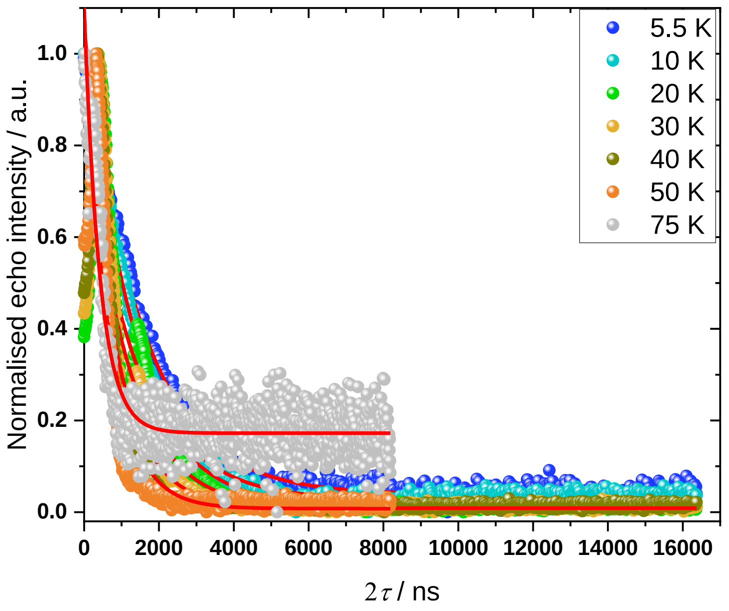

Figure S20. Normalised Hahn echo intensities (scatter) as a function of 2τ measured for **0.5%** with $B_0 \| C_3$ at the $(1 \leftrightarrow 2)_{\|}$ transition at different temperatures. The best fit to a mono exponential is shown as red lines.

Table S4. T_1 values in μs obtained by fitting the inversion recovery measurements shown in Figures S3-S9 to either a mono-exponential or bi-exponential. For the complexes fitted with a biexponential two T_1 values are given, one corresponding to the fast process and one corresponding to the slow process.

	$\left\|\frac{5}{2}\right\rangle \rightarrow \left\|\frac{7}{2}\right\rangle$	$\left\|\frac{3}{2}\right\rangle \rightarrow \left\|\frac{5}{2}\right\rangle$	$\left\|\frac{1}{2}\right\rangle \rightarrow \left\|\frac{3}{2}\right\rangle$	$\left\|-\frac{1}{2}\right\rangle \rightarrow \left\|\frac{1}{2}\right\rangle$	$\left\|-\frac{3}{2}\right\rangle \rightarrow \left\|-\frac{1}{2}\right\rangle$	$\left\|-\frac{5}{2}\right\rangle \rightarrow \left\|-\frac{3}{2}\right\rangle$	$\left\|-\frac{7}{2}\right\rangle \rightarrow \left\|-\frac{5}{2}\right\rangle$
5.5 K	72(2)<br>863(17)	35.1(7)<br>1845(14)	32(1)<br>1521(13)	472(10)<br>3507(64)	37(1)<br>2169(23)	43(1)<br>2478(25)	117(2)<br>2573(32)
10 K	45(3)<br>384(15)	-	-	100(2)<br>645(7)	-	-	66.8(6)<br>469(3)
20 K	19(1)<br>73(6)	-	-	12.4(2)<br>77.2(4)	-	-	18.4(7)<br>61(2)
30 K	-	-	-	6.87(3)<br>31.7(1)	-	-	9.5(2)<br>25.8(8)
40 K	4.3(1)<br>14.1(1)	3.43(4)<br>10.8(3)	3.30(3)<br>11.7(1)	3.28(2)<br>12.3(1)	3.40(3)<br>12.4(1)	1.20(1)<br>12.1(5)	4.3(2)<br>11(1)
50 K	1.7(4)<br>4.2(7)	-	-	1.68(2)<br>5.82(4)	-	-	2.33(7)<br>6.0(4)
75 K	-	-	-	1.43(1)	-	-	1.04(1)
100 K	-	-	-	0.70(1)	-	-	-
125 K	-	-	-	0.39(1)	-	-	-

Table S5. T_m values in ns obtained by fitting the Hahn echo measurements shown in Figures S10-S17 to a mono-exponential.

	$\left\|\frac{5}{2}\right\rangle \rightarrow \left\|\frac{7}{2}\right\rangle$	$\left\|\frac{3}{2}\right\rangle \rightarrow \left\|\frac{5}{2}\right\rangle$	$\left\|\frac{1}{2}\right\rangle \rightarrow \left\|\frac{3}{2}\right\rangle$	$\left\|-\frac{1}{2}\right\rangle \rightarrow \left\|\frac{1}{2}\right\rangle$	$\left\|-\frac{3}{2}\right\rangle \rightarrow \left\|-\frac{1}{2}\right\rangle$	$\left\|-\frac{5}{2}\right\rangle \rightarrow \left\|-\frac{3}{2}\right\rangle$	$\left\|-\frac{7}{2}\right\rangle \rightarrow \left\|-\frac{5}{2}\right\rangle$
5.5 K	1096(19)	1249(5)	1375(7)	1298(8)	1402(5)	1515(6)	1553(8)
10 K	-	1062(10)	1261(9)	1212(9)	1244(7)	1278(8)	1309(6)
20 K	862(21)	-	-	1292(29)	-	-	1419(37)
30 K	-	-	-	1062(22)	-	-	1136(30)
40 K	570(17)	668(9)	764(9)	676(13)	593(5)	691(13)	873(24)
50 K	483(15)	-	-	319(5)	-	-	706(20)
75 K	-	-	-	140(1)	-	-	421(11)
100 K	-	-	-	103(2)	-	-	-
125 K	-	-	-	62(4)	-	-	-

Gd(trensal) 10^{-3} % with $B_0 \perp C_3$

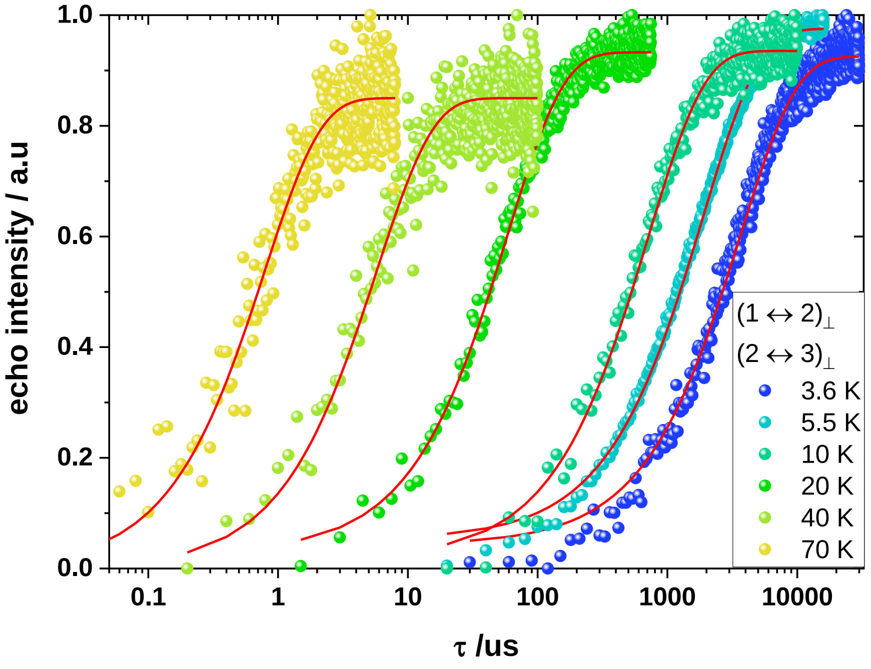

Figure S21. . Normalised echo intensities of an inversion recovery sequence (scatter) as a function of τ measured for **10^{-3} %** with $B_0 \perp C_3$ at the$(1\leftrightarrow2)_\perp$ transition at different temperatures. The best fit to a mono exponential is shown as red lines.

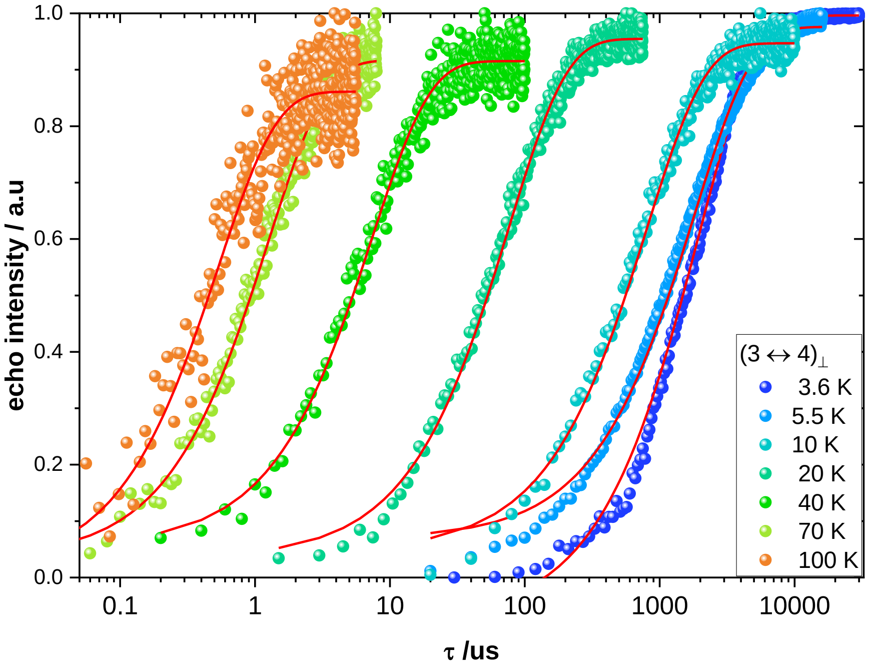

Figure S22. . Normalised echo intensities of an inversion recovery sequence (scatter) as a function of τ measured for **10^{-3} %** with $B_0 \perp C_3$ at the$(3\leftrightarrow4)_\perp$ transition at different temperatures. The best fit to a mono exponential is shown as red lines.

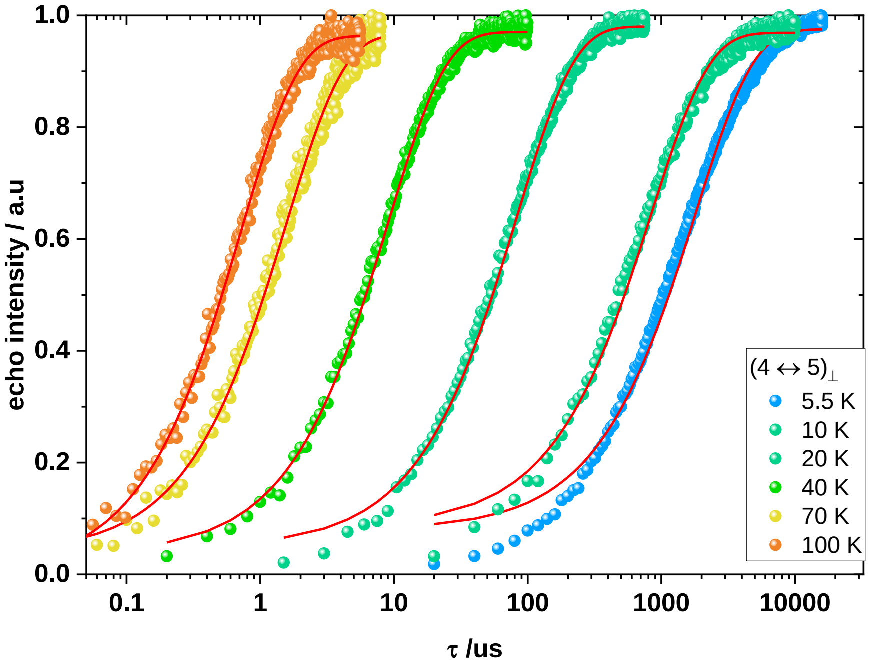

Figure S23. . Normalised echo intensities of an inversion recovery sequence (scatter) as a function of τ measured for 10^{-3} % with $B_0 \perp C_3$ at the$(4 \leftrightarrow 5)_\perp$ transition at different temperatures. The best fit to a mono exponential is shown as red lines.

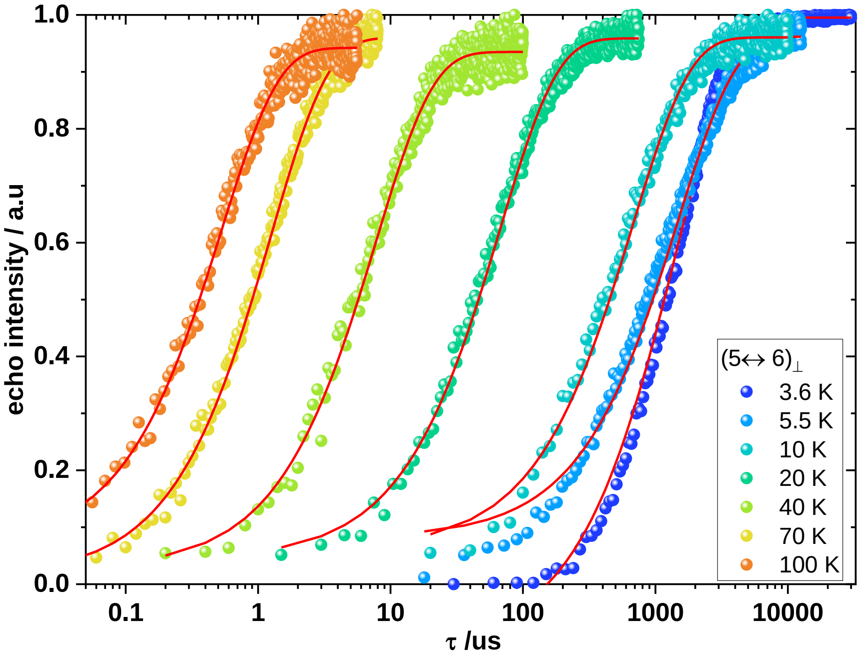

Figure S24. . Normalised echo intensities of an inversion recovery sequence (scatter) as a function of τ measured for 10^{-3} % with $B_0 \perp C_3$ at the$(5 \leftrightarrow 6)_\perp$ transition at different temperatures. The best fit to a mono exponential is shown as red lines.

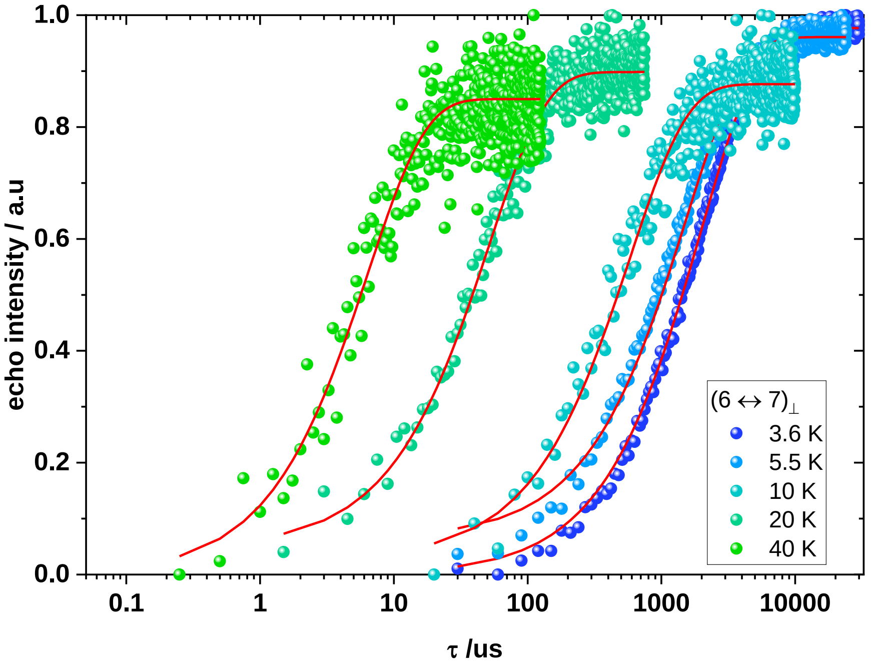

Figure S25. . Normalised echo intensities of an inversion recovery sequence (scatter) as a function of τ measured for **10**$^{-3}$ % with $B_0 \perp C_3$ at the$(6\leftrightarrow7)_\perp$ transition at different temperatures. The best fit to a mono exponential is shown as red lines.

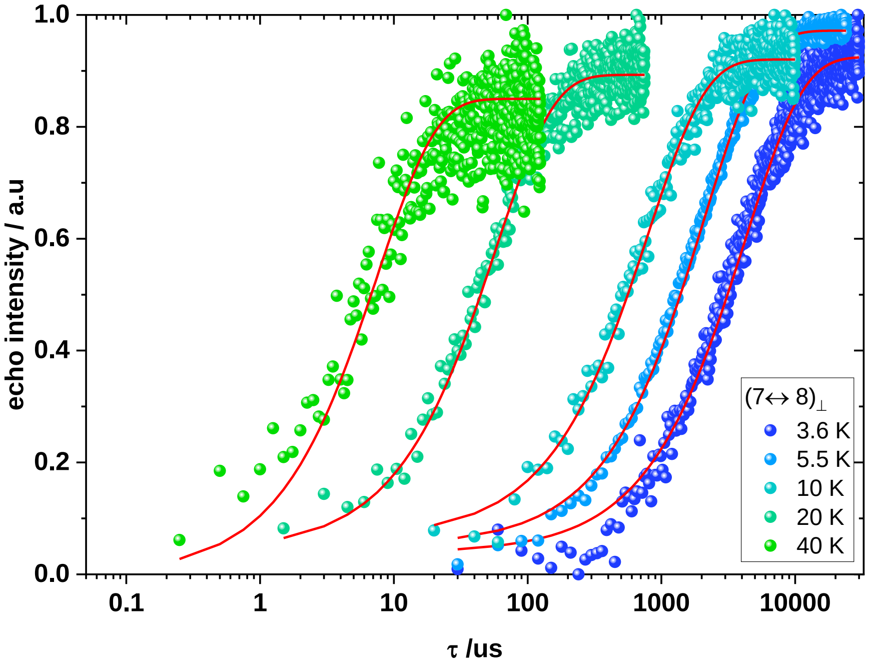

Figure S26. . Normalised echo intensities of an inversion recovery sequence (scatter) as a function of τ measured for **10**$^{-3}$ % with $B_0 \perp C_3$ at the$(7\leftrightarrow8)_\perp$ transition at different temperatures. The best fit to a mono exponential is shown as red lines.

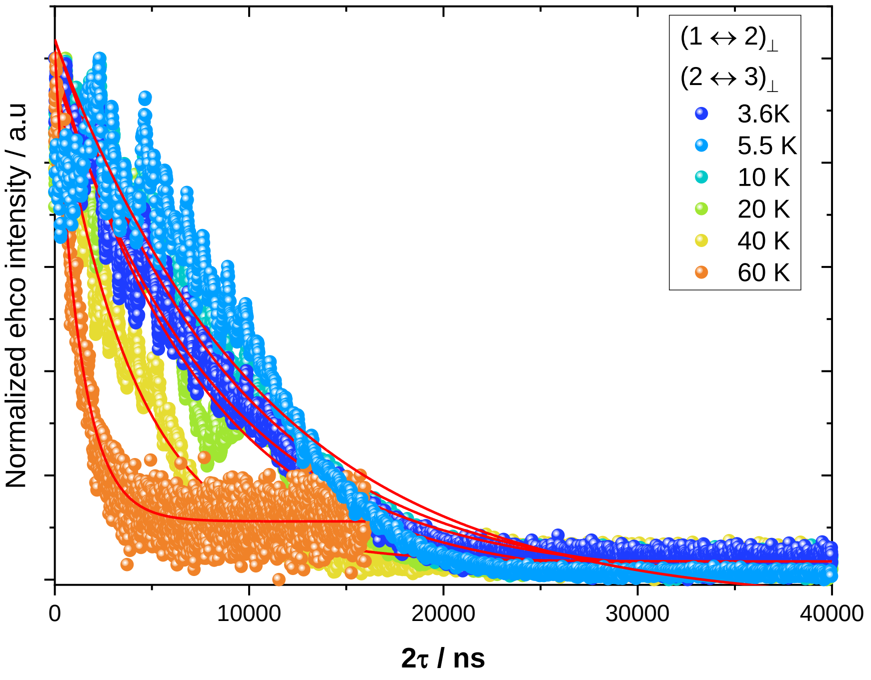

Figure S27. Normalised Hahn echo intensities (scatter) as a function of τ measured for 10⁻³ % with $B_0 \perp C_3$ at the (1↔2)⊥ transition at different temperatures. The best fit to a mono exponential is shown as red lines.

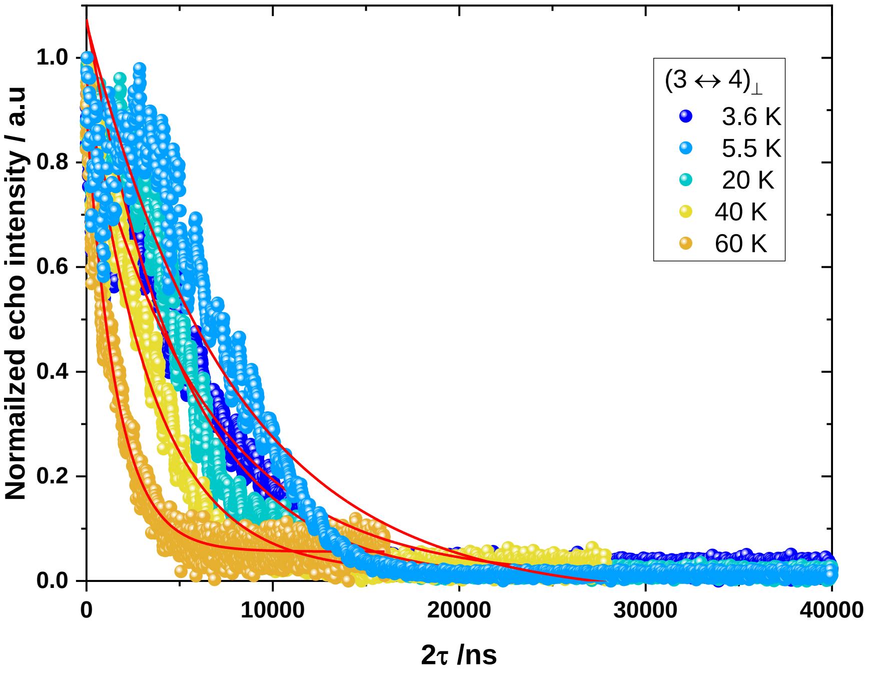

Figure S28. Normalised Hahn echo intensities (scatter) as a function of τ measured for 10⁻³ % with $B_0 \perp C_3$ at the (3↔4)⊥ transition at different temperatures. The best fit to a mono exponential is shown as red lines.

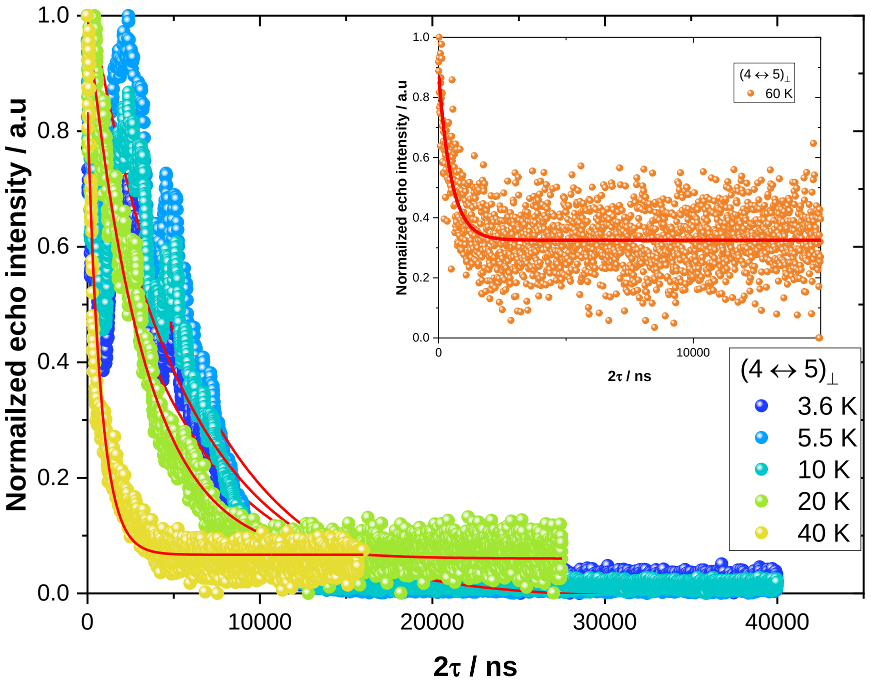

Figure S29. Normalised Hahn echo intensities (scatter) as a function of τ measured for **10⁻³ %** with $B_0 \perp C_3$ at the $(4\leftrightarrow5)_{\perp}$ transition at different temperatures. The best fit to a mono exponential is shown as red lines.

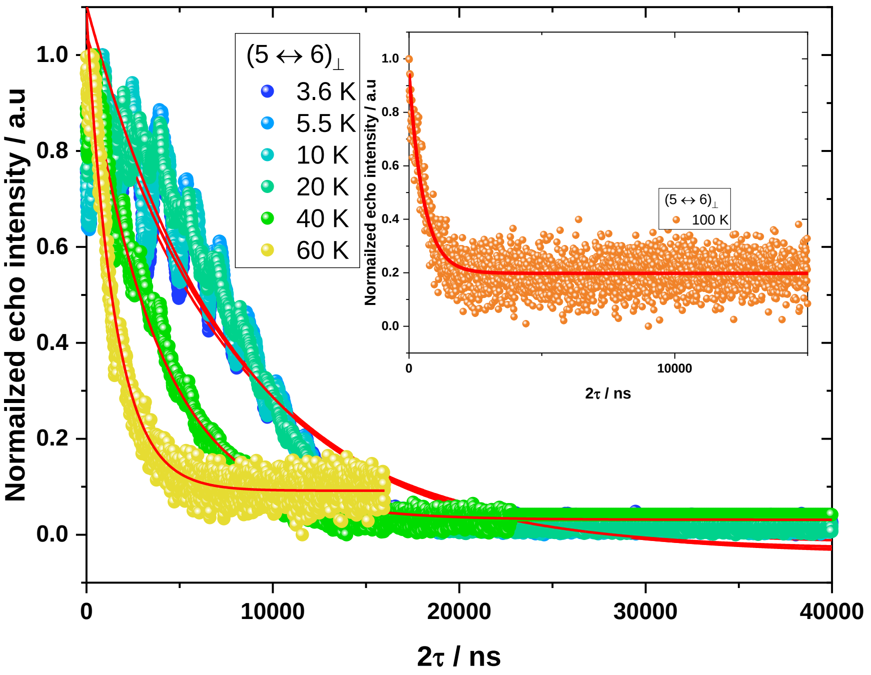

Figure S30. Normalised Hahn echo intensities (scatter) as a function of τ measured for **10⁻³ %** with $B_0 \perp C_3$ at the $(5\leftrightarrow6)_{\perp}$ transition at different temperatures. The best fit to a mono exponential is shown as red lines.

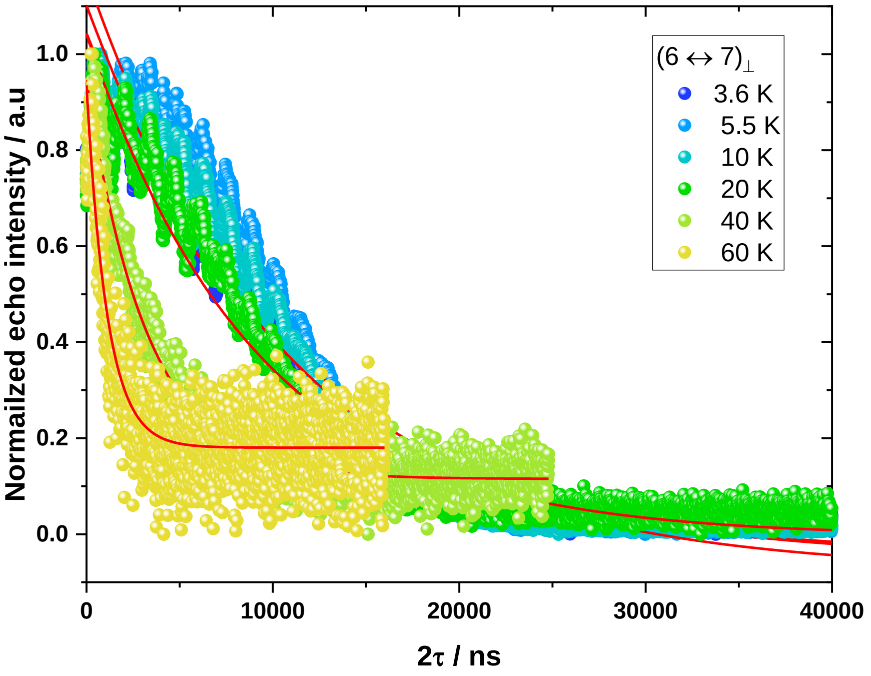

Figure S31. Normalised Hahn echo intensities (scatter) as a function of τ measured for 10⁻³ % with $B_0 \perp C_3$ at the $(6\leftrightarrow 7)_\perp$ transition at different temperatures. The best fit to a mono exponential is shown as red lines.

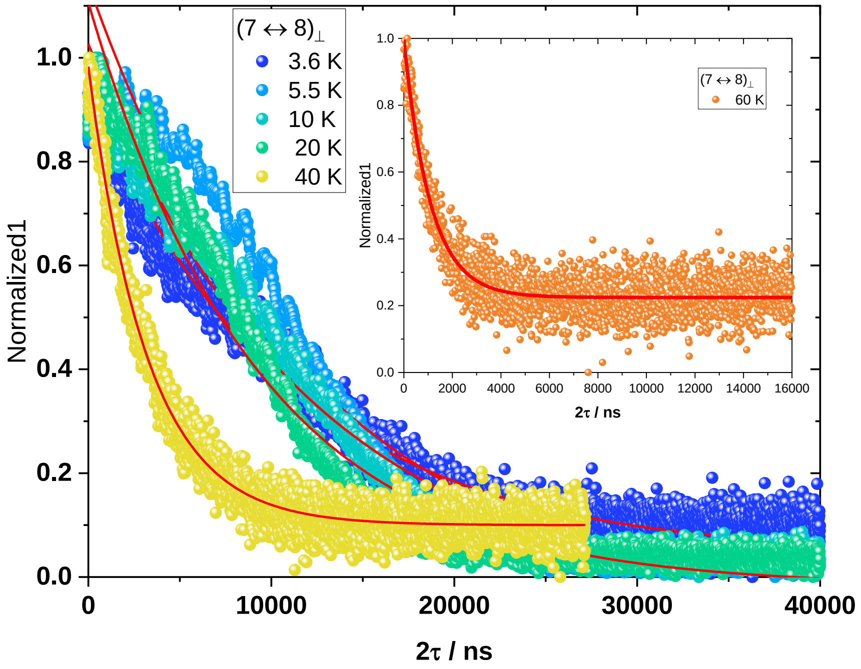

Figure S32 Normalised Hahn echo intensities (scatter) as a function of τ measured for 10⁻³ % with $B_0 \perp C_3$ at the $(7\leftrightarrow 8)_\perp$ transition at different temperatures. The best fit to a mono exponential is shown as red lines.

Short refocusing pulse

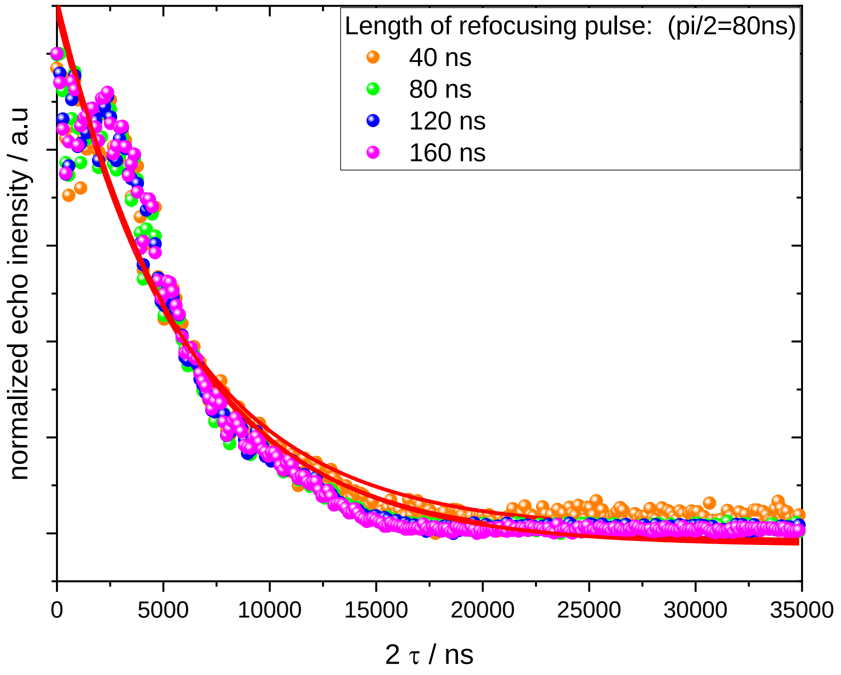

Figure S33 Variable refocusing pulse length for **10⁻³ %** with $B_0 \perp C_3$ at the(3↔4)⊥ transition measured at 5.5 K.

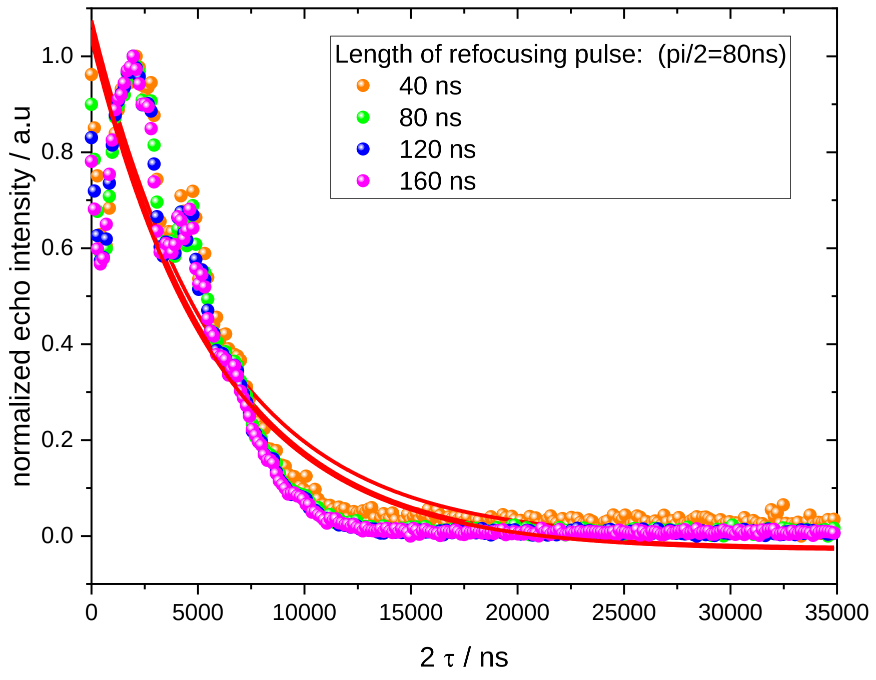

Figure S34 Variable refocusing pulse length for **10⁻³ %** with $B_0 \perp C_3$ at the (4↔5)⊥ transition measured at 5.5 K.

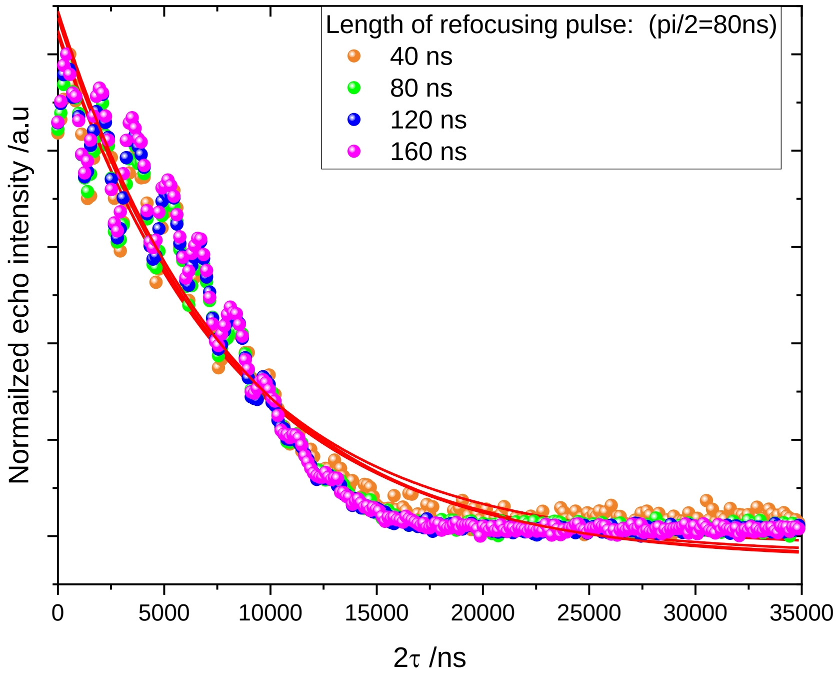

Length of refocusing pulse: (pi/2=80ns)
40 ns
80 ns
120 ns
160 ns
Normailzed echo intensity /a.u
0
5000
10000
15000
20000
25000
30000
35000
2τ /ns

Figure S35 Variable refocusing pulse length for **10^{-3} %** with $B_0 \perp C_3$ at the$(5\leftrightarrow6)_\perp$ transition measured at 5.5 K.

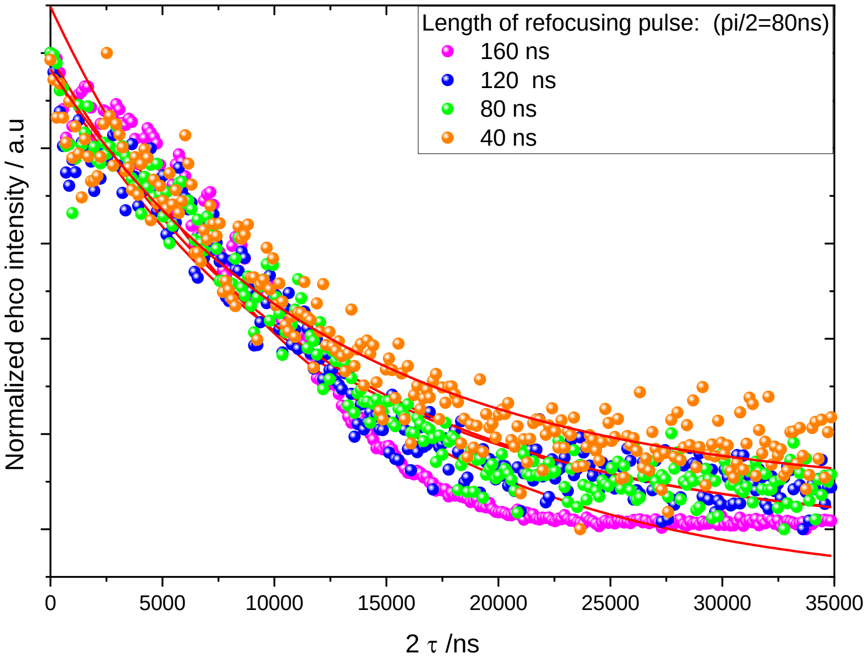

Figure S36 Variable refocusing pulse length for **10^{-3} %** with $B_0 \perp C_3$ at the$(6\leftrightarrow7)_\perp$ transition measured at 5.5 K.

CPMG

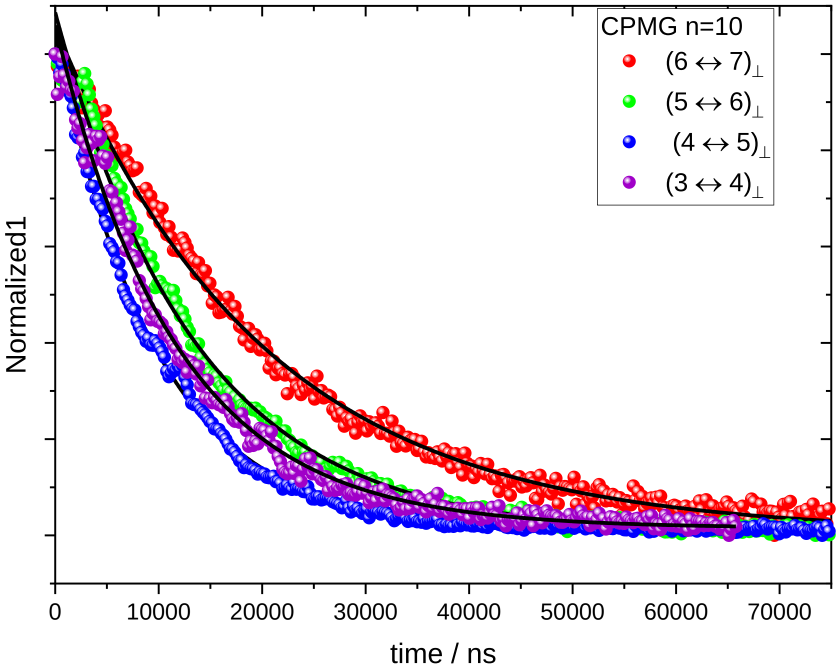

Figure S37 Timetrace of CPMG with a pulse train length of 10 for different transitions, measured for **10**$^{-3}$ **%** with $B_0 \perp C_3$ at 5.5 K.

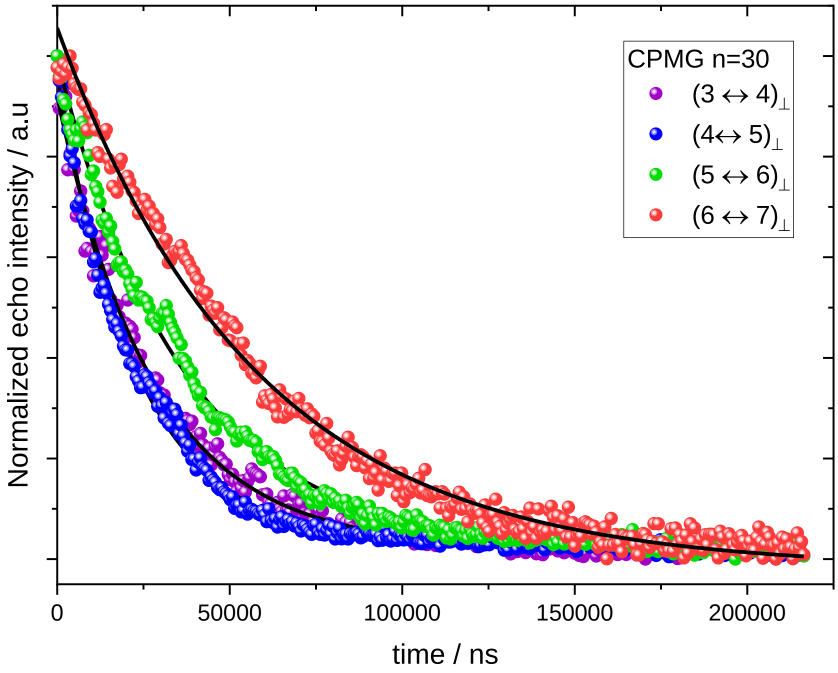

Figure S38 Timetrace of CPMG with a pulse train length of 30 for different transitions, measured for **10**$^{-3}$ **%** with $B_0 \perp C_3$ at 5.5 K.

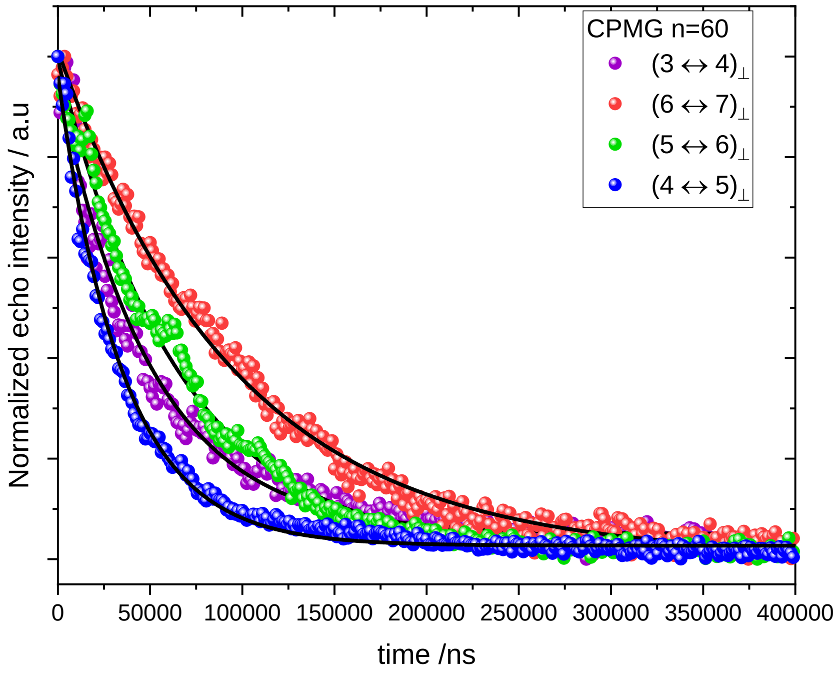

Figure S39 Timetrace of CPMG with a pulse train length of 60 for different transitions, measured for **10^{-3} %** with $B_0 \perp C_3$ at 5.5 K.

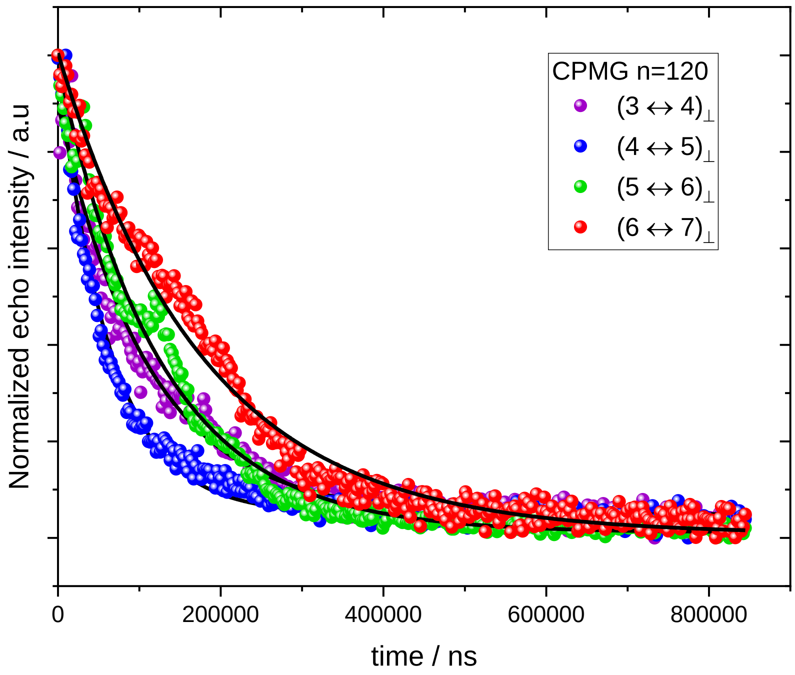

Table S6. T_m values for **10^{-3} %** with $B_0 \perp C_3$ at 5.5 K extracted from CPMG of various pulse train lengths.

	$(3 \leftrightarrow 4)_\perp$ /ns	$(4 \leftrightarrow 5)_\perp$ /ns	$(5 \leftrightarrow 6)_\perp$ /ns	$(6 \leftrightarrow 7)_\perp$ /ns
0	6452	5841	8167	10802
10	11588	9632	13548	20087
30	27926	23270	36498	57198
60	50617	35254	65853	95604
120	109601	64516	124820	177607

Rabi oscillations

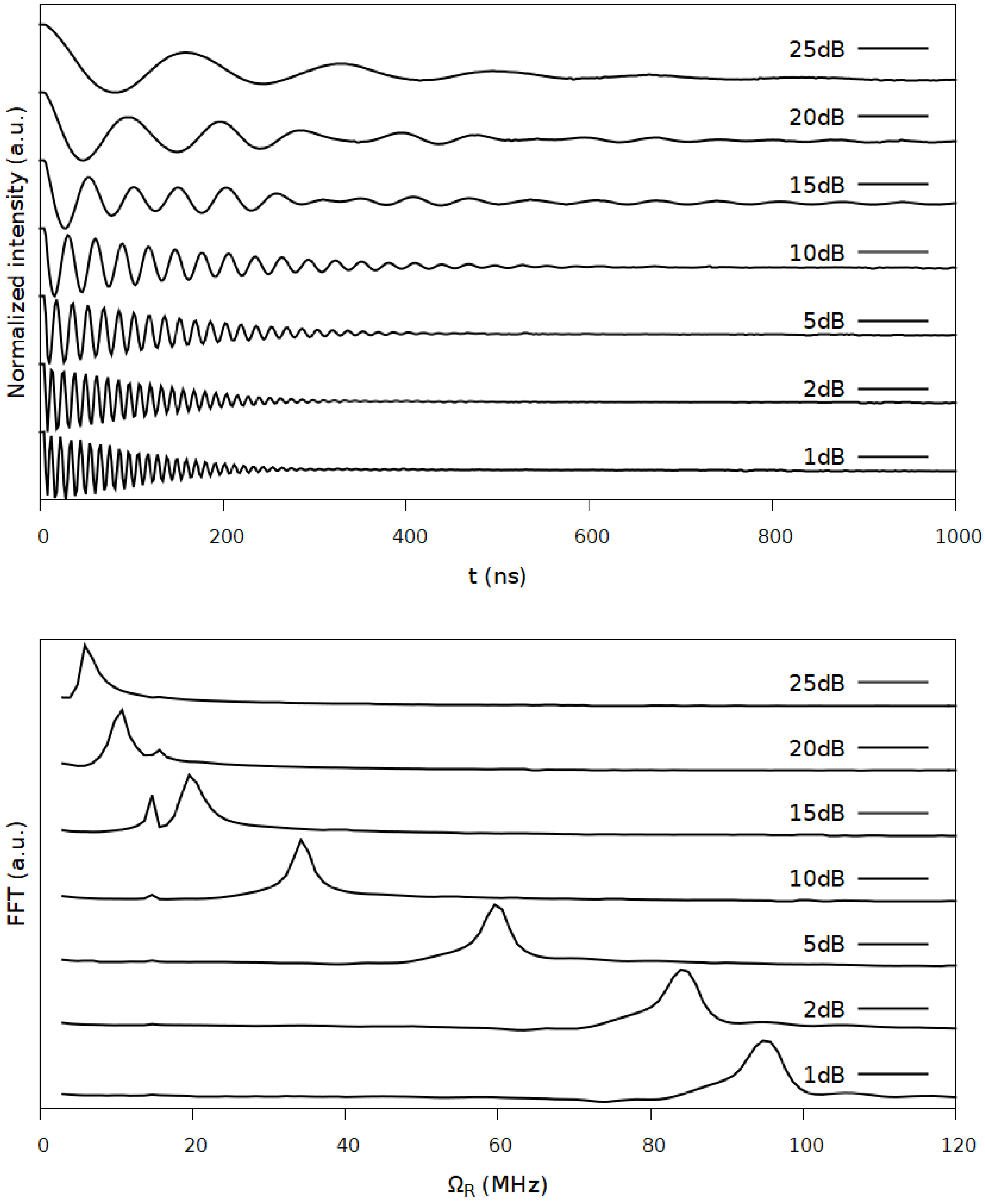

Figure S40. Rabi Oscillations measured for **0.5 %** with $B_0 \| C_3$ at the $(4 \leftrightarrow 5)_{\parallel}$ transition and 30K with varying microwave attenuation (top) and correspond Fourier transforms (bottom). The extra peaks at around 15 MHz corresponds to the Larmor frequency of ^{1}H at this field.

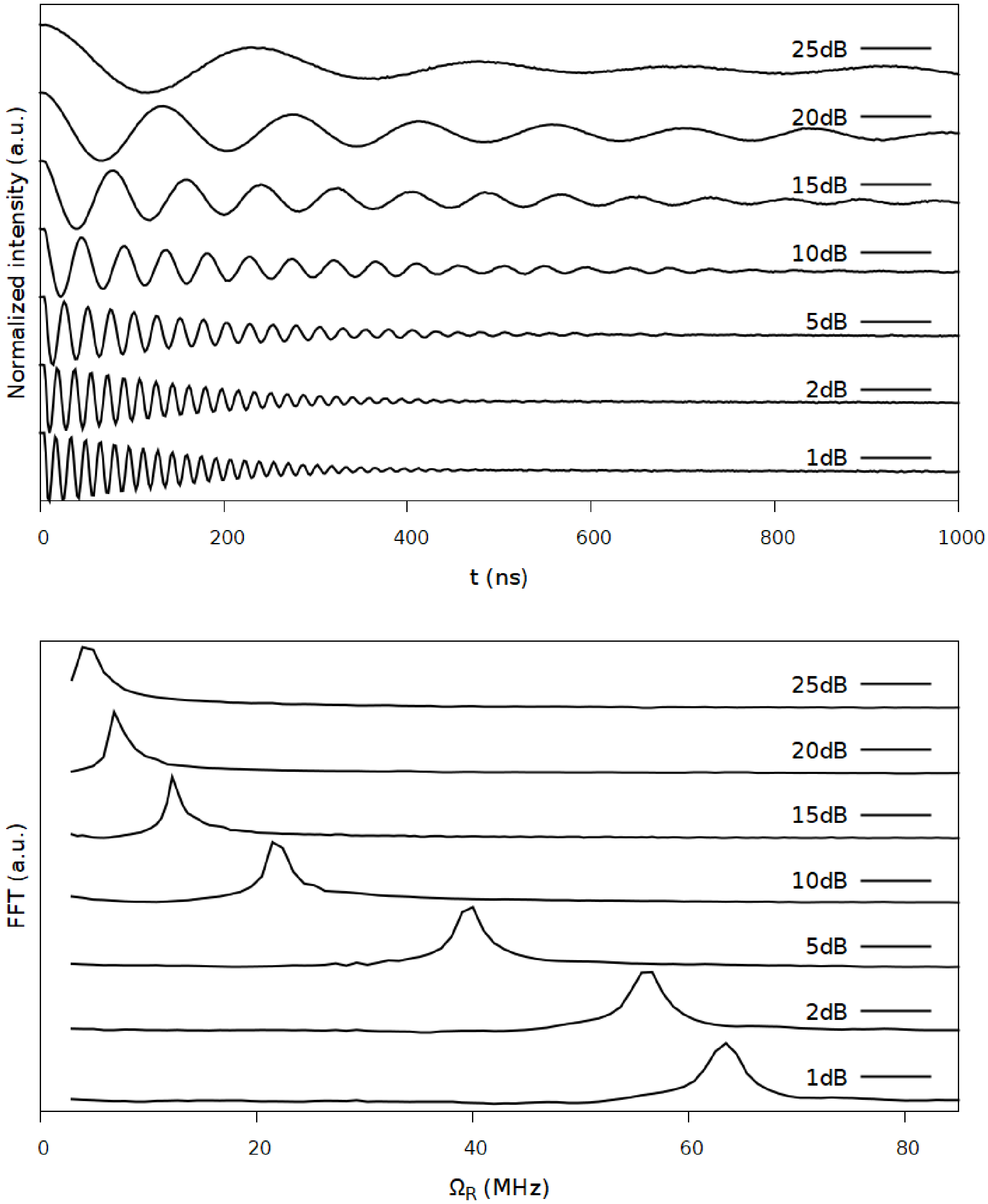

Figure S41. Rabi Oscillations measured for **0.5** % with $B_0 \| C_3$ at the $(1 \leftrightarrow 2)_{\|}$ transition and 30K with varying microwave attenuation (top) and correspond Fourier transforms (bottom). The extra small peaks at around 30 MHz corresponds to the Larmor frequency of ^1H at this field.

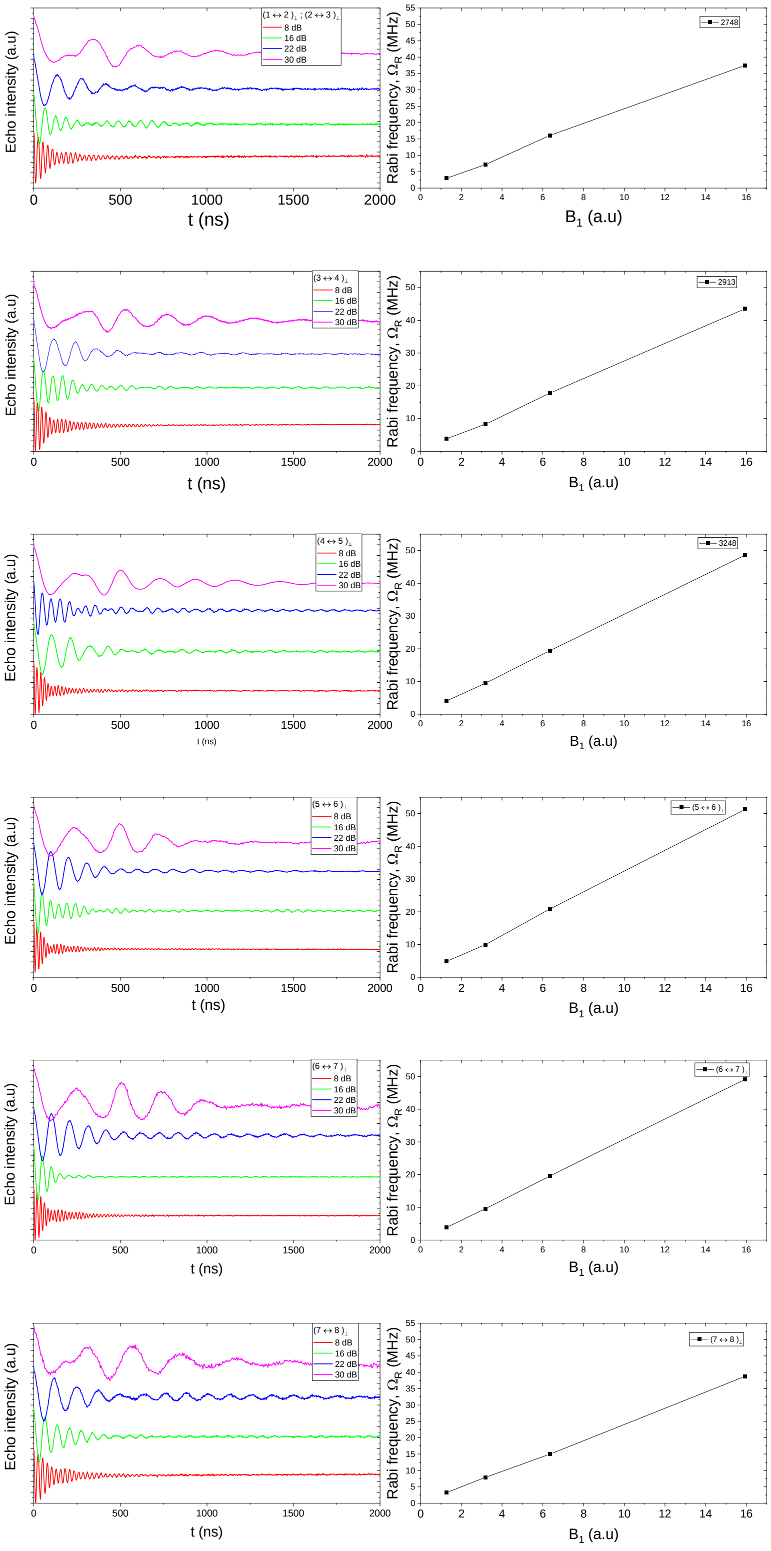

Figure S42. Rabi Oscillations of 10^{-3} % with $B_0 \perp C_3$ measured at all the allowed transions at 5K with varying microwave attenuation and correspond Fourier transforms.

References

(1) Buch, C. D.; Kundu, K.; Marbey, J. J.; van Tol, J.; Weihe, H.; Hill, S.; Piligkos, S. Spin–Lattice Relaxation Decoherence Suppression in Vanishing Orbital Angular Momentum Qubits. *J. Am. Chem. Soc.* **2022**. https://doi.org/10.1021/jacs.2c07057.
